# Hydraulics and Structural Mechanics Jointly Shape Root‐to‐Leaf Scaling of Xylem Conduit Traits

**DOI:** 10.1111/pce.15660

**Published:** 2025-06-05

**Authors:** Milos Simovic, Sean T. Michaletz

**Affiliations:** ^1^ Department of Botany The University of British Columbia Vancouver British Columbia Canada; ^2^ Biodiversity Research Centre The University of British Columbia Vancouver British Columbia Canada

**Keywords:** bootstrapping, hydraulic model, plant allometry, power law, scaling, thickness‐to‐span ratio, tip‐to‐base xylem widening, xylem resistance

## Abstract

Xylem conduit morphology is shaped by the challenges of minimizing hydraulic resistance and preventing conduit wall collapse during vertical sap transport. While hydraulic theories predict that conduits widen from tip to base to minimize resistance, theory has not addressed how collapse prevention influences vertical variation in conduit morphology. Additionally, scaling relationships in roots remain largely unexplored. Here, we evaluate existing theories for conduit diameter scaling and synthesize new theory for vertical variation in thickness‐to‐span ratios. We test these theories using a novel bootstrapping approach to minimize sampling biases and analyze a data set of nearly 600 000 xylem conduits spanning above‐ and belowground organs from five conifer species. As predicted, conduits widened with distance from the leaf tip, with scaling exponents closely aligning with theoretical predictions. Conduits also widened from fine roots to coarse roots, mirroring aboveground patterns. Thickness‐to‐span ratios increased from base to tip and consistently exceeded the predicted critical collapse limit. These findings reveal how the physics of sap transport shape xylem morphology to balance hydraulic efficiency and structural stability. By combining novel theory, robust statistical methods, and comprehensive data, this study refines scaling predictions and advances understanding of mechanisms shaping xylem anatomy across plant organs.

## Summary statement

We examine how hydraulic and structural mechanics jointly shape xylem conduit morphology. We synthesize extant theory for resistance minimization with novel theory for wall collapse from sap tension gradients, and test predictions using a dataset of nearly 600 000 conduits spanning leaf to root tips.

## 1 Introduction

Water transport through plants is tightly linked to the carbon assimilation rates and microclimates of leaves and canopies, influencing plant‐atmosphere feedbacks and driving vegetation responses to climate change (Blonder et al. 2023; Garen et al. 2023; Michaletz and Garen 2024). Understanding how xylem morphology balances the competing demands of water transport and structural integrity is essential for predicting plant functioning and adaptation in a changing climate (Baum et al. in review). Vertical sap transport imposes two key selective pressures on xylem morphology: hydraulic resistance and the risk of conduit wall collapse (embolism risk also influences xylem morphology but is not the focus here; Hacke et al. 2001; Tyree and Zimmermann 2002). In vascular plants, selection favors xylem traits that maintain efficient sap transport while preserving structural stability through periods of varying water availability (Niklas 1992, 1997).

The first selective pressure arises from the increase in hydraulic resistance with xylem path length, which limits the efficiency of long‐distance sap transport. The hydraulic resistance, *r* (MPa s m^−^^3^), for a single cylindrical conduit is described by the Hagen–Poiseuille equation

(1) $$r=\;\frac{|\Delta P|}{Q}=\frac{128\eta l}{{\pi d}^{4}},$$

where *∆P* (MPa) is the pressure difference between the ends of the conduit, *Q* (m^3^ s^−^^1^) is the volumetric flow rate, *η* (MPa s) is the viscosity of the sap, *l* (m) is the conduit length, and *d* is the conduit diameter (m; Tyree and Zimmermann 2002). Equation (1) shows that resistance increases linearly with conduit length. For a series of conduits, the total resistance, *R* (MPa s m^−^^3^), is the sum of the resistances of individual conduits

(2) $$R=\sum_{i=1}^{n}r_{i}$$

where *r*_*i*_ (MPa s m^−3^) is the resistance of an individual conduit *i* (Equation 1), from the terminal tip (*i* = 1) to the base (*i* = *n*).

Equation (1) also shows that resistance varies inversely with the fourth power of conduit diameter. Consequently, the total resistance *R* is highly sensitive to the scaling profile of conduit diameters along the network, which is characterized by

(3) $$d=d_{0}L^{\alpha },$$

where *d* (µm) is lumen (conduit) diameter, *d*_*0*_ (µm m^−*b*^) is a normalization constant, *L* (m) is the distance from the tip of the plant, and *α* (dimensionless) is the path length‐scaling exponent. If conduit diameters remained constant along the hydraulic path, *R* would increase linearly with *L* (Equations 1 and 2; Olson et al. 2021). In tall plants, such an increase in resistance would necessitate high sap tensions capable of causing air seed embolisms and reducing the hydraulic conductivity of the stem or branch (Sperry et al. 1988; Tyree and Sperry 1989; Tyree and Zimmermann 2002). Instead, conduit diameters increase from tip to base, which is hypothesized to be a general adaptive response that minimizes resistance to vertical sap transport (West et al. 1999; Koçillari et al. 2021; Olson et al. 2021). We also note that minimizing resistance may not be adaptive in all contexts (Price et al. 2012), and in some cases, increasing resistance may improve fitness (e.g., limiting water loss from fine roots during drought; Cuneo et al. 2016, 2021).

Several hydraulic models make quantitative predictions for *α* (Table 1). The pipe model assumes that conduit diameter remains constant across the path (*α* = 0; Shinozaki et al. 1964a, 1964b). In contrast, the West‐Brown‐Enquist (WBE) model predicts *α* = ¼ based on a hierarchical branching network of tapering conduits optimized to minimize hydraulic resistance while maintaining constant resistance across *L* (West et al. 1999). The height‐corrected WBE model revises this prediction to *α* ≈ ⅕ by accounting for conduit widening as a function of *L* to reflect tree height (Anfodillo et al. 2006; Olson et al. 2018, 2021). The packed conduit model predicts *α* = ½ by incorporating conduit branching and packing (Savage et al. 2010), while the carbon cost‐gain model predicts *α* = ⅙ by optimizing for net carbon gain rather than hydraulic resistance (Hölttä et al. 2011). Finally, the widened pipe model predicts *α* = ¼ near the plant tip, with deviations near the base driven by trade‐offs between hydraulic resistance and construction costs (Koçillari et al. 2021). Empirical data for aboveground organs yielded *α* ≈ 0.24 (Koçillari et al. 2021), consistent with the WBE and widened pipe models. Although conduits are known to widen beyond the stem base into structural and coarse roots (Petit et al. 2009; Prendin et al. 2018; Lintunen and Kalliokoski 2010; Wang et al. 2022), conduit–path length scaling across the entire root network (including fine roots) remains unexplored. This represents a critical knowledge gap, as fine roots are essential for water uptake and transport (McCormack et al. 2015) and play a central role in whole‐plant hydraulic function.

**Table 1:** Predicted path‐scaling exponents, *α* (Equation 1), and diameter‐scaling exponents, *β* (Equiation 4), from several theories for scaling of xylem conduit morphology.

Model	Predicted path‐scaling exponent, *α*	Predicted diameter‐scaling exponent, *β*	Source
Pipe model	0	0	Shinozaki et al. (1964a)
WBE model	¼ = 0.25	⅙ ≈ 0.17	West et al. (1999)
Height‐corrected WBE model	≈ 0.2	≈ 0.11	Anfodillo et al. (2006)
Packed conduit model	½ = 0.5	⅓ ≈ 0.33	Savage et al. (2010)
Carbon cost‐gain model	⅙ ≈ 0.17	np	Hölttä et al. (2011)
Widened pipe model	¼ = 0.25*	np	Koçillari et al. (2021)

Abbreviation: np, no prediction.

* Deviates ~30% from power law near base of plant.

Scaling of conduit diameters can also be expressed in terms of the external diameter of plant organs such as stems and roots. Since the WBE model and its extensions (Table 1) assume that *L* ∝ *D* ^*2/3*^ across the entire branching network (Supporting Information S1: Appendix S1; Greenhill 1881), Equation (3) can be rewritten as

(4) $$d=d_{0}D^{\beta },$$

where *d*_*0*_ (µm m^−*b*^) is the normalization constant, *D* (m) is the external organ diameter, and *β* (dimensionless) is the diameter‐scaling exponent. These models thus make quantitative predictions for *β* (Table 1). For example, the WBE model predicts *β* = ⅙ (West et al. 1999), the height‐corrected WBE model predicts *β* ≈ 0.11, and the packed conduit model predicts *β* = ⅓ (Savage et al. 2010). For tree stems, an empirical estimate of *β* ≈ 0.36 (95% CI: 0.32–0.39; Olson and Rosell 2013) is consistent with the packed conduit model but not with other models (Table 1). In roots, scaling between conduit diameter and external diameter has been seldom explored (but see Biondini 2008), and there is no evidence that *β* agrees with predictions from any of the models in Table 1.

A second selective pressure on xylem morphology is the risk of conduit collapse due to wall stresses induced by tension in the water column. The cohesion‐tension theory posits that transpiration creates surface tension forces in the leaf's evaporative surfaces that are transmitted through a continuous water column by cohesion, adhesion, and tension forces, effectively pulling water from the soil into the roots and through the xylem to the leaves (Dixon and Joly 1894, 1895; Tyree and Zimmermann 2002; Angeles et al. 2004). Sap tension transmitted via adhesion to conduit walls induces bending stresses that can cause wall collapse (Hacke et al. 2001), reducing the hydraulic conductivity of the organ (Michaletz et al. 2012) and limiting stomatal conductance and photosynthesis rates. In severe cases, this hydraulic bottleneck can lead to desiccation and death of distal organs (Tyree et al. 1994; Rood et al. 2000; Sperry et al. 2002 Michaletz et al. 2012). Conduit wall collapse can be characterized as bending of a rectangular plate (Young et al. 2012; Hacke et al. 2001), such that

(5) $$P_{\mathrm{crit}}=\frac{\sigma }{\beta }{\left(\frac{t}{b}\right)}^{2},$$

where *P*_crit_ (MPa) is the critical collapse pressure, *σ* (MPa) is the modulus of rupture, *β* (dimensionless) is a coefficient that varies with the span‐to‐length ratio, *t* (µm) is the thickness of the double conduit wall, and *b* (µm) is the span of the same conduit wall. The thickness‐to‐span ratio (*t*/*b*)^2^ (dimensionless) quantifies the ability of the conduit wall to resist collapse. The vulnerability of conduit walls to collapse under extreme conditions can be assessed using the safety factor, which is the ratio of the observed thickness‐to‐span ratio to the critical value (Hacke et al. 2001).

While the mechanics of conduit wall collapse have been well described (Hacke et al. 2001), their potential role in driving vertical variation in thickness‐to‐span ratios remains unexplored. Variation in thickness‐to‐span ratios has been reported for three tree species (Domec et al. 2009; Prendin et al. 2018), but no mechanistic hypotheses have been proposed to explain this pattern. Here, we propose for the first time that vertical variation in sap tension, combined with conduit diameter variation, co‐drives the critical thickness‐to‐span ratio required to prevent collapse. As water potential decreases from roots to leaves, reaching its lowest value in the leaf airspaces proximal to the stomata (Buckley and Sack 2019), conduit walls must be sufficiently reinforced to withstand the extreme sap tensions experienced, particularly at higher positions. This hypothesis predicts a negative relationship between thickness‐to‐span ratio and path length *L*, with greater reinforcement near the tip where sap tensions are highest. Consistent with this hypothesis are observations in some angiosperm species, where conduits near the plant tip possess narrower perforations and perforation plates with wider “collars” that may provide increased mechanical support against high sap tensions (Olson et al. 2020).

In this study, we examine how xylem conduit morphology reflects the physical demands of sap transport, including both flow resistance and structural stability. We evaluate competing theories that predict scaling exponents *α* and *β* (Table 1) by analyzing empirical scaling relationships between conduit diameter *d*, path length *L*, and organ diameter *D*. Additionally, we synthesize and test mechanistic theory that predicts vertical variation in thickness‐to‐span ratios from first principles, predicting that (*t*/*b*)^2^ decreases with *L* while remaining sufficiently high at all points along the hydraulic path to prevent conduit wall collapse. To achieve these goals, we used a semi‐automated approach to measure traits of 600 000 xylem conduits in 188 tissue samples spanning the full leaf‐to‐root axis. The total number of conduits measured in our data set is up to 100 times greater than in previous studies. We analyze this unprecedented data set using a novel bootstrapping approach, which ensures robust parameter estimation and addresses biases inherent in xylem morphology datasets. Together, these approaches provide exceptional resolution for testing mechanistic theories and advancing our understanding of xylem structure‐function relationships across plant organs.

## 2 Materials and Methods

### 2.1 Plant Material

In May and June of 2021, we sampled 5–6 individuals from each of five conifer species: *Callitropsis nootkatensis* (D. Don) Oerst., *Picea sitchensis* (Bong.) Carr., *Thuja plicata* Donn ex D. Don, *Tsuga heterophylla* (Raf.) Sarg., and *Tsuga mertensia* (Bong.) Carr. (Supporting Information S1: Table S1). We selected the most common conifers inhabiting the temperate rainforests of British Columbia, which are nevertheless absent from or underrepresented in global datasets (Koçillari et al. 2021). The individuals were located at The University of British Columbia Botanical Garden (49.25383, −123.24731) and the Capilano Watershed (49.46189, −123.04511), both near Vancouver, British Columbia, Canada. From each individual, we sampled one terminal branch with intact leaves and twigs ( ~1 m in length), one coarse root with intact fine roots ( ~ 0.5 m in length), and one stem increment core ( ~5 cm in length; Supporting Information S1: Figure S1). Terminal branches were excised using a pole pruner and accessed from a tree canopy walk or ladder. Unlike for leaves and stems, we did not sample along the longest root axis due to the difficulty and destructiveness of excavation. Increment cores were sampled just above the root collar using a 5.15 mm I.D. increment borer (Haglöf). Cores were immediately placed in centrifuge tubes containing 40% ethanol to inhibit microbial growth (Gärtner and Schweingruber 2013). All samples were then transported to the laboratory, where the branches and roots were sectioned into 10 cm segments, placed in 50 mL centrifuge tubes containing 40% ethanol, and stored at 4°C. The path length *L* from leaf tip to each sampling point was measured using a meter tape or laser hypsometer (Forestry Pro II, Nikon). We employed the 2‐point tangential method for measuring branch height, which accurately estimates vertical distances in non‐leaning trees growing on flat terrain (Larjavaara and Muller‐Landau 2013; Simovic et al. 2024).

Samples were sectioned to enable measurement of xylem morphological traits (Supporting Information S1: Figure S2). Following best practices (von Arx et al. 2016), we obtained 20 µm transverse cross sections from leaves, twigs, the base of sampled terminal branches, stem cores, coarse roots (outside diameter ≥ 3 mm), fine roots (5th–3rd order roots; McCormack et al. 2015), and very fine roots (1st order roots). Root orders were classified according to the Horton‐Strahler scheme (Horton 1945; Strahler 1957). Fully intact, healthy leaves from terminal buds were sectioned at the midpoint of the leaf. Twigs were sectioned a few millimeters below the terminal bud (in *Picea* and *Tsuga* individuals) or apicalmost bud (in *Callitropsis* and *Thuja* individuals, which lack an obvious terminal bud). Branches were sectioned 1–2 cm from the proximal end. Tree cores were mounted onto wooden dowels using clear waterproof gel glue (Clear Grip, The Gorilla Glue Company) and sectioned transversely (Wegner et al. 2013). While our sampling was not as densely spaced near the plant tip as recommended for estimating conduit widening in aboveground organs of individual plants (see Olson et al. 2021 for sampling guidelines), our goal was to characterize general patterns in conduit morphology along the entire hydraulic path from fine roots to leaves across individuals. Our approach therefore involved sampling a single position per segment and is appropriate given the large number of conduits measured at each *L* (Simovic and Michaletz 2025). Coarse roots were sectioned near the proximal end. Fine roots were sectioned halfway between their junction with coarse roots and 2nd and 1st order fine roots, whereas very fine roots were sectioned near the root cap. Branches, stem cores, and coarse roots were sectioned using a sliding microtome (AO 860, American Optical). Leaves, fine roots, and very fine roots were first mounted in a 4% agarose solution (low melting‐point agarose, BioBasic) and then sectioned using a vibrating blade microtome (VT1000 S, Leica Biosystems).

Fresh sections were immediately stained with 0.8% safranin and mounted onto microscope slides. Sections were observed under a light microscope (Olympus BX51WI) at various magnifications depending on the size of the xylem conduits (100× for branches, coarse roots, and cores, 200–400× for leaves, fine and very fine roots, and twigs). For shoots containing only primary xylem (leaves, twigs) we photographed a minimum of five vascular bundles per sample, and for fine roots we photographed the entire vascular cylinder in each sample. For organs containing mostly secondary xylem (branches, stem cores, and coarse roots), we scanned across the growth rings and took a minimum of ten photographs per sample.

### 2.2 Image Analysis of Xylem Morphological Traits

We measured xylem conduit diameters, cell wall thicknesses, and thickness‐to‐span ratios (Supporting Information S1: Figure S2) using ROXAS version 3.0.1 (von Arx and Carrer 2014) in the ImagePro Plus software (version 6.3; Media Cybernetics, Rockville, MD, USA). Cell wall thickness was measured for the two radial and two tangential cell walls bordering the conduit (Prendin et al. 2017), and the smaller of these values (i.e., the wall most susceptible to collapse; Equation 5) was used to calculate the thickness‐to‐span ratio. Where applicable, we aggregated data from several outer rings (Olson et al. 2018), as our focal trees grew under stable environmental conditions with little interannual variation in climate or water availability (Petit 2024; Prendin et al. 2018). Under such conditions, anatomical differences across recent rings are minimal, and combining them does not bias scaling relationships. However, when comparing individuals across environmental gradients (e.g., moisture availably), data should be taken from the outermost ring to avoid confounding effects of axial conduit widening and variable growth rates (Petit 2024). Each image was carefully inspected for undetected conduits and “pseudo‐conduits” (e.g., resin duct epithelial cells) that the software had erroneously identified as conduits. Additionally, because our focal species are tracheid‐bearing conifers, the ROXAS data were not subject to vessel measurement errors known in some angiosperms, such as the accidental inclusion of imperforate tracheary elements (Olson 2023). Following automated analysis, missing conduits were manually traced and added to the data set, and pseudo‐conduits were manually deleted. Six images were excluded due to cutting artefacts (von Arx et al. 2016). In total, we analyzed 1020 images containing 580 049 xylem conduits from 188 tissue samples.

To characterize the mean conduit diameter of each tissue sample, we calculated the hydraulic diameter (*d*_*h*_; µm) as

(6) $$d_{h}=\frac{\sum_{i=1}^{n}d_{i}^{5}}{\sum_{i=1}^{n}d_{i}^{4}},$$

where *d*_*i*_ (µm) is the diameter of the *i*th conduit in each sample (Sperry et al. 1994). Unlike the arithmetic mean, *d*_*h*_ gives more weight to larger, earlywood xylem conduits, which contribute disproportionately more to the overall hydraulic conductivity despite being far less numerous than latewood vessels (Sperry et al. 1994).

### 2.3 Modeling the Critical Collapse Limit of Xylem Conduits

We simulated the critical collapse limit of xylem conduits along the hydraulic path using models for water potential gradients in the soil‐plant‐atmosphere continuum (Lechthaler et al. 2020) and for bending of a rectangular plate supported on four edges (Young et al. 2012, p. 532). First, we used Model II standardized major axis (SMA) regression to fit a linearized form of Equation (3) to our data using the sma function in the smatr package (Warton et al. 2012). The linearized form is obtained by log‐transforming Equation (3) to give

(7) $$\log_{10}(d)=d+\alpha \log_{10}(L).$$

SMA regression is appropriate for quantifying power law relationships (Warton et al. 2006) as it minimizes residual variation in both the *x* and *y* dimensions rather than in *y* only as in Model I ordinary least squares (OLS) regression. The resulting regression equation was used to predict variation in *d* in 100 µm increments along *L* from leaves to the base of the tree. As a first approximation, the hydraulic path of each tree was modelled as a series of vertically stacked pipes that gradually widen from the tips of the leaves to the base of the tree (Lechthaler et al. 2020; Olson et al. 2021). The pressure head due to gravity (approximately 0.01 MPa per meter height) is relatively small for the tree heights in this study (e.g., 0.3 MPa at the tip of the tallest tree) and was therefore excluded from the model; however, it should be considered for taller trees. The resistance *r* of each segment was calculated using Equation (1).

Volumetric sap flow rate *Q* (m^3^ s^−^^1^) through the hydraulic path was calculated using Darcy's law (Reid et al. 2005)

(8) $$Q=\frac{\Delta \Psi_{s,l}}{R},$$

where *∆Ψ*_*s,l*_ (MPa) is the difference in water potential between the soil (*ψ*_soil_; MPa) and leaf (*ψ*_leaf_; MPa), and *R* (MPa s m^−^^3^) is the sum of the individual resistances of all segments (given by Equation 2). From here, the difference in water potential between *ψ* of segment *n* and *ψ*_leaf_ (*∆ψ*_*n,l*_ = *ψ*_*n*_ −* ψ*_*l*_, MPa) was calculated as

(9) $$\Delta \psi_{n,l}=Q\sum_{i=1}^{n}r_{i},$$

where *r*_*i*_ is the resistance of segment *i*. *ψ*_leaf_ was taken −4.7 MPa, which is the minimum *ψ* measured from July 2022 to July 2023 in these species (Simovic and Michaletz unpublished manuscript). *ψ*_soil_ was taken as −1.5 MPa, which is a typical wilting point (Tolk 2003). The water potential for any given segment, *i* (dimensionless), along the hydraulic path (*ψ*_*i*_; MPa) was then calculated as

(10) $$\psi_{i}=\psi_{\mathrm{leaf}}+\Delta \psi_{n,l}.$$

Water potential influences sap tension, *P*_*i*_ (MPa), where $P_{i}=\psi_{i}$, which determines the mechanical stresses conduits must withstand. The critical thickness‐to‐span ratio, (*t/b*)^2^_crit_ (dimensionless), at which wall collapse occurs for a given sap tension was calculated by rearranging Equation (5) (Hacke et al. 2004; Young et al. 2012, p. 532), such that

(11) $${\left(\frac{t}{b}\right)}_{\text{crit}}^{2}{=P}_{i}\frac{\beta }{\sigma },$$

where *σ* (MPa) is the modulus of rupture for green cell wall material. *β* (dimensionless) is a bending stress constant that depends on the ratio of conduit wall span (*b*) to length (*l*); it approximates 0.25 when *b*/*l* ≤ 0.5, which is always the case in xylem conduits (Sperry et al. 2006). We obtained estimates of *σ* for each species (Forest Products Laboratory 2021) and used the mean value (41.6 MPa) to simulate a general critical collapse limit. To complement this, we also simulated species‐specific critical collapse limits using each species' value of *σ*. Both general and species‐specific critical collapse limits were estimated by numerically solving Equations ((1), (2)) and ((7), (8), (9), (10), (11)) in 100 µm increments along *L*.

Earlywood and latewood conduits often exhibit substantially different critical thickness‐to‐span ratios due to their differing lumen diameters. To distinguish between them, we used Mork's index, *M* (dimensionless), defined as

(12) $$M=\;\frac{{\text{4x}}_{\tan}}{d_{\text{rad}}},$$

where *x*_tan_ (µm) is the mean tangential single‐cell wall thickness (averaged across inner and outer tangential walls), and *d*_rad_ (µm) is the radial lumen diameter (Mork 1928; Denne 1988). The factor of 4 accounts for the duality and double thickness of adjacent cell walls. ROXAS calculates *M* using tangential wall thickness instead of radial wall thickness due to the latter's sensitivity to artifacts such as pit chamber widening (von Arx and Carrer 2014). Cells with *M* > 1 are classified as latewood, while *M* ≤ 1 corresponds to earlywood. This approach aligns well with other methods for identifying earlywood and latewood transitions, such as threshold density and inflection point methods (Antony et al. 2012).

### 2.4 Statistical Analyses

All scaling relationships were estimated using SMA fits of Equations ((3), (4)) with the sma function in the smatr package (Warton et al. 2012), as well as OLS fits using base R. We fitted models to both aggregated data to estimate general scaling relationships (sensu Olson et al. 2021), and to individual tree data to evaluate within‐species variation in scaling exponents (Supporting Information S1: Appendix S2). We also evaluated alternative models (exponential, logarithmic, piecewise, and quadratic) and compared their fits to power laws using the Akaike Information Criterion (AIC; Supporting Information S1: Appendix S3). For piecewise regressions, we used the segmented function in R to fit linear models with breakpoints. To test for curvature in log‐log space, we fit quadratic models (Pawar et al. 2016; Price et al. 2022). A quadratic coefficient *X* ^*2*^ greater than 0 indicates upward curvature, while *X* ^*2*^ < 0 indicates downward curvature. Differences in slope estimates between organs were tested using the slope.test function in smatr. We also used piecewise regression to examine variation in *d* and *(t/b)* ^*2*^ among organs spanning the root‐to‐leaf axis. To achieve this, we treated organs (a categorical variable) as a continuous variable by ordering them according to their axial position, that is, from leaves (#1) to fine roots (#7). To fit SMA regressions to (*t*/*b*)^2^ data, we used relative path length (*L/H*) rather than absolute path length (*L*), as sap tension depends on both tree height and position along the hydraulic path (Lechthaler et al. 2020). Using *L/H* standardizes path length across trees of different heights, facilitating meaningful comparisons of (*t*/*b*)^2^ scaling relationships among individuals.

Because models could not be fitted between *d* and *L* or *d* and *D* in the full data set due to the unequal number of conduits sampled across *L* and *D* (Supporting Information S1: Figures S3 and S4), we used subsampling and bootstrapping to resample the data set before model fitting (Simovic and Michaletz 2025; Maitner et al. 2023). First, we divided the raw, log‐normally distributed data set into equally spaced logarithmic bins, using both *L* and *D* as the binning variables. We ensured that each bin had at least *n* = 100 observations, setting the number of bins so that the bin with the fewest observations met this minimum. For the subsampling approach, we determined which *L* and *D* bin has the lowest number of raw conduit diameter observations (*n*_*d*,min_). We then sampled, without replacement, *n*_*d*,min_ observations from each of the bins. For the bootstrapping approach, we first split the data into equally spaced logarithmic bins and determined which *L* and *D* bin has the highest number of raw conduit diameter observations (*n*_*d,*max_). We then randomly sampled, with replacement, *n*_*d*,max_–*n*_*d*,bin_ observations from each bin, where *n*_*d*,bin_ is the number of raw conduit diameter observations in each bin. The bootstrapped data were then combined with the raw data, yielding a sample size of *n*_*d*,max_ in each bin. Given that SMA slope and confidence interval estimates will vary stochastically between resampled datasets, both the subsampling and bootstrapping procedures were repeated for a set number of iterations (1–50 000 for subsampling and 1–1000 for bootstrapping). The maximum number of iterations was set by re‐running the resampling and fitting procedures until the slopes and confidence intervals remained stable with additional iterations (Supporting Information S1: Figures S5– S10). Unless otherwise stated, all reported model fits used bootstrapped data.

All statistical analyses were performed in *R* (R Core Team 2024), and the code for replicating all the analyses and figures is available from the Open Science Framework at https://doi.org/10.17605/OSF.IO/FTR4M.

## 3 Results

**Figure 1:**
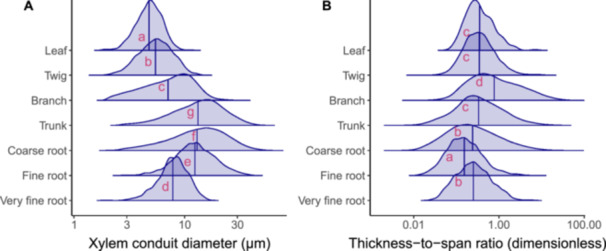
Frequency distributions of (A) xylem conduit diameters *d* and (B) thickness‐to‐span ratios (*t*/*b*)^2^ for different organs along the hydraulic path. Data were combined from all individuals (*N* = 27) and species (*N* = 5). Vertical lines represent mean values of each distribution. Red letters indicate significant differences between mean values for different organs (Tukey's HSD test with Šidák adjustment for multiple pairwise comparisons). Both xylem conduit diameters and thickness‐to‐span ratios were log_10_‐transformed to meet ANOVA assumptions and aid visualization.

Frequency distributions of xylem conduit diameters and thickness‐to‐span ratios for different organs along the hydraulic path, from fine roots to leaves, are shown in Figure 1. Across all species and individuals, xylem conduit diameters ranged from 1.62 to 70.79 µm (Figure 1A) while thickness‐to‐span ratios varied from 0.0024 to 90.65 (Figure 1B). Mean conduit diameter increased from the tips of leaves (4.76 µm) to the base of the trunks (13.19 µm) and decreased from trunk bases to the tips of fine roots (7.82 µm; *p* < 2.0 × 10^−^^16^; Figure 1A). Mean thickness‐to‐span ratio increased from leaves to branches (*p* < 2.0 × 10^−^^16^) where it reached its apex (mean (*t*/*b*)^2^ = 0.78), and progressively decreased from branches to the trunks (mean (*t*/*b*)^2^ = 0.33) and roots (*p* < 2.0 × 10^−^^16^; mean (*t*/*b*)^2^ = 0.15–0.25; Figure 1B).

**Figure 2:**
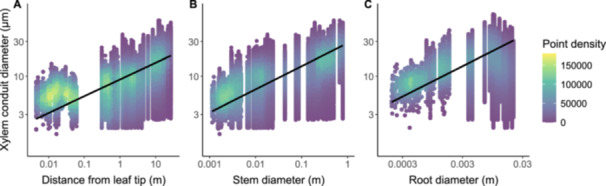
Scaling of xylem conduit diameters along the hydraulic path length and with external stem and root diameter. (A) Relationship between conduit diameter and distance from leaf tip (*α* = 0.23, 95% confidence interval (CI) = 0.23–0.23, *p* = 2.22 × 10^−^^16^, *r* ^*2*^ = 0.27). (B) Relationship between conduit diameter and external stem diameter (*β* = 0.32, 95% CI = 0.32–0.32, *p* = 2.22 × 10^−^^16^, *r* ^*2*^ = 0.47). (C) Relationship between conduit diameter and external root diameter (*β* = 0.42, 95% CI = 0.42–0.42, *p* = 2.22 × 10^−^^16^, *r* ^*2*^ = 0.19). [Color figure can be viewed at wileyonlinelibrary.com]

The relationship between xylem conduit diameter and distance from the leaf tip is shown in Figure 2A. SMA analyses showed that conduit diameters increased with distance from the leaf tip following *α* = 0.23 (*p* = 2.22 × 10^−^^16^, *r* ^*2*^ = 0.27), with extremely tight 95% confidence intervals (CIs; 0.23–0.23) that statistically excluded all values predicted by hydraulic models (Table 1). Similar results were observed in OLS analyses, with consistently positive but sometimes shallower slopes (Supporting Information S1: Table S2). The relationship exhibited upward curvature in log‐log space (quadratic coefficient *L* ^*2*^ = 0.07). Our empirical estimates of *α* did not differ significantly (*p* = 0.40) between the subsampled and bootstrapped datasets (i.e., non‐averaged diameter data, *d*). However, both differed significantly (*p* = 0.01 and 0.005, respectively) from *α* = 0.20 (95% CI: 0.18–0.22) obtained by fitting averaged data (i.e., hydraulic diameter, *d*_*h*_; Supporting Information S1: Figure S11), and from the mean *α* = 0.20 (95% CI: 0.19–0.22; Supporting Information S1: Figure S12) calculated by fitting data for each individual and averaging the resulting exponents.

The relationship between xylem conduit diameter and external stem diameter is shown in Figure 2B. Conduit diameters scaled with external stem diameter following *β* = 0.32 (*p* = 2.22 × 10^–^^16^, *r* ^*2*^ = 0.47; Figure 2B), again with extremely tight 95% CI (0.32–0.32) that was nearly identical to the value of *β* = ⅓ predicted by the packed conduit model and excluded all other model predictions (Table 1). The relationship between conduit diameter and external stem diameter exhibited slight upward curvature in log‐log space (*D*_stem_^*2*^ = 0.02). In roots, conduit diameters scaled with external root diameter following *β* = 0.42 (*p* = 2.22 × 10^−^^16^, *r* ^*2*^ = 0.19; Figure 2C), with extremely tight 95% CI (0.42–0.42) that excluded all values predicted by theory (Table 1). In contrast to stems, the relationship between conduit diameter and external root diameter exhibited slight downward curvature in log‐log space (*D*_root_^*2*^ = −0.20). The organ diameter‐scaling exponent *β* was significantly greater in roots than in stems (*p* < 1.11 × 10^−^^16^). Furthermore, the normalization constant *d*_*0*_ was significantly higher for roots (*d*_*0*_ = 2.19) than for stems (*d*_*0*_ = 1.45; *p* < 1.11 × 10^−^^16^). Finally, our empirical estimates of *β* for both stems and roots did not differ (*p* = 0.63 and 0.60, respectively) between the subsampled and bootstrapped datasets (i.e., non‐averaged diameter data, *d*). However, stem and root *β* for subsampled data were significantly different (*p* = 0.007 and *p* = 0.001, respectively; Supporting Information S1: Figures S13 and S14) from for hydraulic diameter data. Stem and root *β* estimated for bootstrapped data were also significantly different (*p* = 0.008 and *p* = 0.001, respectively) from *β* for hydraulic diameter data.

**Figure 3:**
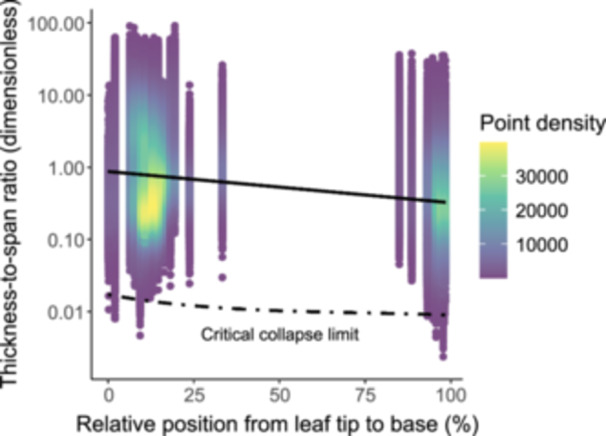
Thickness‐to‐span ratios of xylem conduits and the critical conduit collapse limit along the hydraulic path for five conifers. The critical collapse limit (dashed black line) is a numerical solution of the model for xylem conduit wall collapse in response to vertical sap tension gradients (Equations. 1‐2, 5, 7‐11), calculated in 100 µm increments along the hydraulic path of each tree. The *x*‐axis represents the relative position along the hydraulic path, with 0% corresponding to the leaf tip and 100% corresponding to the base of the tree. The thickness‐to‐span ratios of nearly all conduits (99.96%) exceeded the critical limit, while the safety factor of 99.60% of conduits was greater than 2, indicating that xylem walls are generally overbuilt relative to the critical collapse limit. These findings underscore the importance of resistance to collapse as a key constraint on xylem conduit morphology. [Color figure can be viewed at wileyonlinelibrary.com]

Model simulations (Equations (7), (8), (9), (10), (11)) predicted a threefold decrease in the critical collapse limit (*t*/*b*)^2^_*crit*_ from the tip (*L/H* ≈ 0%) to the base (*L/H* ≈ 100%), ranging from 0.028 to 0.009 (Figure 3). Consistent with this prediction, observed thickness‐to‐span ratios (*t*/*b*)^2^ exhibited a significant exponential decrease across relative position from leaf tip to base, *L/H* (*p* < 2.22 × 10^−^^16^; Figure 3). Nearly all conduits (99.96%) had thickness‐to‐span ratios that exceeded the critical collapse limit required to prevent failure under extreme water stress. Of the tiny fraction of conduits predicted to collapse (0.04%), most were located in the earlywood (*n* = 183), with only a few in the latewood (*n* = 3) and primary xylem of leaves and twigs (*n* = 2). Conduit collapse remained exceptionally rare (99.94% of conduits safe) even when modelled separately for each species using species‐specific modulus of rupture values. Across all conduits, the median safety factor was 43.60 (IQR: 18.53–124.04), with 90% of values falling between 6.35 and 541.97. As with (*t*/*b*)^2^ (Supporting Information S1: Table S1), safety factors differed substantially between different types of xylem (Supporting information S1: Table S4).

## 4 Discussion

This study examines how xylem conduit morphology is shaped by the physics of sap transport, striking a balance between hydraulic efficiency and mechanical stability. We assessed predictions from competing theories for scaling of conduit diameters with hydraulic path length (Table 1) and evaluated our novel theory (Equations (7), (8), (9), (10), (11)) for thickness‐to‐span ratios (*t*/*b*)^2^. Although the observed diameter‐length scaling exponents were statistically different than the values predicted by the theories, they were numerically close to several predictions, supporting the hypothesis that xylem morphology evolved to minimize hydraulic resistance with increasing path lengths (Figure 2A). Additionally, we observed that (*t*/*b*)^2^ decreased with relative distance from the tip but consistently exceeded the critical collapse limit in essentially all conduits (Figure 3), emphasizing the importance of structural reinforcement in maintaining xylem integrity.

Scaling relationships between conduit diameter *d* and path length *L* have been modelled for decades, with predictions for scaling exponents *α* varying widely (Table 1). Several models predict exponents with similar magnitudes (West et al. 1999; Anfodillo et al. 2006; Hölttä et al. 2011; Olson et al. 2018, 2021; Koçillari et al. 2021) or identical values (West et al. 1999; Koçillari et al. 2021), despite substantial differences in assumptions, optimization criteria, and mechanistic detail (Supporting Information S1: Appendix S1). Our results for five conifer species show that *d* scales as the 0.23‐power of *L* (Figure 2A). This exponent is nearly identical to the value of *α* = 0.24 observed across a broader range of species and plant functional types (Koçillari et al. 2021), and it is generally consistent with predictions of *α* = ⅕ to ¼ from several models (West et al. 1999; Anfodillo et al. 2006; Olson et al. 2021; Koçillari et al. 2021). Although these models differ in various respects (see Supporting Information S1: Appendix S1), they share a core assumption that cumulative hydraulic resistance is minimized as total path length increases (Supporting Information S1: Appendix S1).

Notably, however, our data reveal curvature in log‐log space for tip‐to‐base conduit widening, contradicting the strict power‐law behavior predicted by most models (Table 1). This curvature is most pronounced in the uppermost meter of the trees, near the tip (Figure 2A; Supporting Information S1: Table S3). Here, widening occurs less rapidly than expected based on power law expectations from theory (Table 1) and empirical exponents calculated when fitting averaged (i.e., hydraulic diameter) data (Supporting Information S1: Figure S11A). These findings suggest that fixed‐exponent scaling may oversimplify patterns of conduit widening and underscore the need for models that accommodate variable scaling behavior along the hydraulic path (Koçillari et al. 2021).

The scaling of conduit diameter *d* with distance from tip *L* can also be expressed in terms of stem diameter *D*_stem_, assuming a consistent scaling relationship between *L* and *D*_*stem*_ from tip to base (e.g., *L* ∝ *D*_stem_^⅔^; Greenhill 1881; Rosell et al. 2017). We found that *d* scaled with outside stem diameter *D*_stem_ as *β* ≈ 0.32, which is nearly identical to the *β* = ⅓ predicted by the packed conduit model (Savage et al. 2010). The packed conduit model predicts *β* = ⅓ and *α* = ½, the latter prediction being simply an expression of the former under the assumption that *L* scales as the ⅔‐power of *D*_stem_ (see Supporting Information S1: Appendix S1; Savage et al. 2010).

Why did our results support the packed conduit model's (Savage et al. 2010) prediction of *β* = ⅓, but not its *α* = ½ prediction? The most likely explanation is that *L* does not consistently scale as the ⅔‐power of stem diameter within individual trees (Price et al. 2022). This scaling relationship is typically observed at the whole‐tree level across diverse species and height ranges (Jucker et al. 2022; Simovic and Michaletz 2025), but not within individuals where the exponent can vary substantially from tip to base (McMahon and Kronauer 1976; Bertram 1989). In our study, ⅔‐power scaling was observed exclusively in trunks, not in branches or twigs (Supporting Information S1: Figure S15), which likely reflects the contrasting functional roles of trunks and branches (McMahon and Kronauer 1976; Price et al. 2022). The flow similarity model (Price et al. 2022) offers an alternative prediction that *L* scales as the square of stem diameter across much of the xylem network, with a shift toward ⅔‐power scaling near the trunk. Applying this prediction to the packed conduit model yields a predicted *α* ≈ 0.17, much closer to our observed value of *α* = 0.23 (Figure 2A). Similarly, using our measured species mean (*L ∝ D*_stem_^1.58^) revises the model prediction to *α* ≈ 0.21. These adjusted predictions are more consistent with our empirical results than the original packed conduit model prediction of *α* = ½. This discrepancy may also explain why Olson and Rosell (2013) found support for *β* = ⅓, even though subsequent studies observed poor model fit when *D*_stem_ was expressed as *L* (Koçillari et al. 2021).

While the scaling of aboveground xylem conduit traits with distance along the hydraulic path has been studied for over a century (Sanio 1872; West et al. 1999; Savage et al. 2010; Olson and Rosell 2013), comparatively little attention has been given to belowground organs such as coarse and fine roots (Petit et al. 2009; Lintunen and Kalliokoski 2010; Hacke 2015; Prendin et al. 2018). We found that *d* scaled with *D*_root_ as *β* ≈ 0.42. Larger and more rapidly widening conduits in roots suggest a prioritization of water and nutrient transport efficiency, potentially at the cost of increased vulnerability to air‐seed and freeze‐thaw embolism (Pittermann and Sperry 2003, 2006). However, roots are largely protected from air‐seed embolism due to low sap tensions and buffered against freeze‐thaw embolism by insulating soil and snow cover (Simovic and Michaletz unpublished manuscript). Additionally, since belowground organs typically represent a smaller proportion of total tree biomass than aboveground organs (Niklas 2005; Qi et al. 2019), investing in larger conduits may increase hydraulic conductance per unit biomass and help maintain uniform conductance across the root‐shoot continuum. Finally, we note that our sampling did not extend along the full length of the main root axis, precluding comparison of scaling exponents *α* between roots and stems. This remains a key gap in our understanding of whole‐plant xylem architecture and warrants further investigation.

Extensive evidence shows that while tip‐to‐base conduit widening is a universal feature among terrestrial plants, gymnosperms and angiosperms exhibit distinct patterns (Anfodillo et al. 2006; Prendin et al. 2018; Lechthaler et al. 2020; Koçillari et al. 2021). Gymnosperms typically have lower scaling exponents (*α*) and normalization constants (*d*_*0*_), resulting in conduits (tracheids) that widen more gradually and remain narrower along the hydraulic path than angiosperm conduits (vessels; Anfodillo et al. 2006; Prendin et al. 2018; Lechthaler et al. 2020). In gymnosperms, tracheids fulfill both transport and structural roles, whereas in angiosperms these functions are divided between vessels and fibers. Despite having narrower conduits, gymnosperms likely achieve comparable hydraulic conductivity by allocating a greater proportion of cross‐sectional area to conductive tracheids (Hacke 2015) and by enhancing permeability through traits such as larger margo pores and shorter leaves with increasing height (Anfodillo and Olson 2024). In contrast, angiosperms compensate for their smaller conductive area by producing larger, more rapidly widening conduits (Koçillari et al. 2021) and by increasing permeability via thinner inter‐vessel pit membranes (Anfodillo and Olson 2024).

We observed that essentially all conduits had thickness‐to‐span ratios sufficiently large to prevent wall collapse under the mechanical stresses imposed by extreme sap tensions, as simulated using Equations (1), (2), (5), and ((7), (8), (9), (10), (11)). This finding suggests that the risk of collapse is a key driver shaping conduit morphology, selectively eliminating conduits lacking sufficient reinforcement to withstand extreme drought conditions. Across our data set, conduits had a median safety factor of 43.59, with even the lowest values exceeding the critical collapse limit in 99.96% of cases. This median safety factor is substantially higher than the values of 3.5 to 9.5 previously reported for conifers (Hacke et al. 2001; Hacke et al. 2004; Domec et al. 2009), likely reflecting the high proportion (81%) of latewood conduits in our data set, which had a median safety factor of 62.21 (Supporting Information S1: Table S4). In contrast, earlywood conduits in our data set had a median safety factor of 10.01, which closely aligns with values reported in previous studies. Such concordance is expected, as the range of earlywood (*t*/*b*)^2^ in our study (0.03–0.25) largely overlap with the range of mean values (0.04–0.19) previously reported for gymnosperm tracheids (Hacke et al. 2001; Hacke et al. 2004; Domec et al. 2009).

In our data set (Supporting Information S1: Figure S16), conduits narrower than approximately 10 µm spanned nearly the full range of cell wall thickness, but conduits wider than 10 µm with thin cell walls ( ~1–1.5 µm) were exceptionally rare. This rarity became even more pronounced among conduits exceeding 30 µm in diameter (Supporting Information S1: Figure S16), consistent with findings from Echeverría et al. (2022), who reported a conspicuous absence of thin‐walled vessels larger than 90 µm across 858 woody angiosperm species. These patterns suggest that selection against collapse has shaped conduit morphology by eliminating vulnerable morphologies and promoting additional reinforcement in wider conduits to preserve function under extreme sap tensions. They are also broadly consistent with recent observations that the pressure required to induce substantial loss of hydraulic conductivity tends to decrease from leaf tip to base (Zambonini et al. 2024), where conduits are typically wider and thus more susceptible to collapse. Furthermore, the high safety factors observed here align with broader trends of xylem adaptation to mechanical stress (Hacke et al. 2004). Together, these findings underscore that xylem reinforcement not only prevents collapse but also provides additional structural safety, particularly in wider conduits. This extra reinforcement likely reflects the critical importance of wider conduits for maintaining hydraulic efficiency while withstanding the mechanical challenges imposed by extreme environmental conditions.

## 5 Conclusion

Our study provides strong evidence that xylem conduit anatomy is fundamentally shaped by the physics of sap transport. Conduits widen predictably from leaf tip to stem base, minimizing hydraulic resistance across the path length. However, previous studies examining this scaling relationship often relied on regression models fitted to averaged data, which can bias scaling exponents and reduce statistical power (Simovic and Michaletz 2025). Recent advances in automated image analysis, including artificial intelligence‐based techniques (Katzenmaier et al. 2023), offer promising opportunities to substantially increase the sample size of conduits measured per image, though with important caveats (Olson 2023). These advances could enhance statistical power, leading to more accurate and precise estimates of empirical scaling exponents and enabling detailed exploration of developmental zones within individual plants (Olson et al. 2021). While the former is critical for comparative studies of tip‐to‐base conduit widening, the latter is an empirical priority that could reveal the limits of plant hydraulic development (Olson et al. 2021).

We also observed that thickness‐to‐span ratios decrease slightly from the leaf tip to the stem base along with sap tension but consistently remain above the critical collapse limit. Notably, wide conduits with thin cell walls were nearly absent from our data set, underscoring strong selective pressure against collapse‐prone structures. These findings suggest that the risk of conduit wall collapse has exerted a substantial influence on xylem evolution, as collapse is an extremely costly and generally irreversible form of hydraulic failure (but see Cochard et al. 2004; Brodribb and Holbrook 2005; Zhang et al. 2023 for instances of reversible collapse). Despite this, tension‐induced conduit collapse has received less attention than other forms of hydraulic dysfunction, such as air seed and freeze‐thaw embolism (but see Michaletz et al. 2012). Our hydraulic model represents an initial attempt to simulate critical collapse limits, but future iterations could incorporate varying water potential scenarios, such as those occurring during extreme droughts and heatwaves (Couvreur et al. 2018). These scenarios are becoming increasingly relevant as climate change intensifies the frequency and severity of extreme weather conditions (White et al. 2023; Calvin et al. 2023; Baum et al. in review). Moreover, empirical research on conduit collapse remains limited, particularly outside of leaves, leaving critical gaps in understanding the specific conditions that induce collapse (Polo et al. 2020; Thonglim et al. 2021). Comparative studies integrating experimental data with collapse model predictions are urgently needed to validate and refine existing models, advancing our understanding of this underexplored aspect of xylem physiology.

## Conflicts of Interest

The authors declare no conflicts of interest.

## Supporting information

Supporting Information

## Data Availability

The data that support the findings of this study are openly available from the Open Science Framework at https://doi.org/10.17605/OSF.IO/FTR4M.
