# Supplementary material for: Hydraulics and Structural Mechanics Jointly Shape Root‐to‐Leaf Scaling of Xylem Conduit Traits
Source: Plant Cell Environ. 2025 Jun 5;48(9):6912–23. doi: 10.1111/pce.15660 (PMC12319298; doi:10.1111/pce.15660)
Supplement: Supplementary file 1 — Supporting Information [file PCE-48-6912-s001.docx]

**Supporting Information**

Article title: Hydraulics and structural mechanics jointly shape root-to-leaf scaling of xylem conduit traits

Authors: Milos Simovic and Sean T. Michaletz

Article acceptance date: 22 May 2025

The following Supporting Information is available for this article:

**Appendix S1** Overview of theoretical models predicting the scaling exponents (*α* and *β*) for the relationship between conduit size and path length & organ diameter.

**Appendix S2** Methods for fitting individual tree data to evaluate within-species variation in scaling exponents.

**Appendix S3** Comparing the quality of different statistical model fits for the scaling relationships explored in the study.

**Table S1** Species and individual information (sample sizes, height, leaf length, and mean values for xylem morphological traits).

**Table S2** Slopes and 95% CIs of key scaling relationships in the study, obtained by fitting the data using both standardized major axis (SMA) and ordinary least squares (OLS) regression.

**Table S3** Comparisons of different model fits (power law, logarithmic, exponential, piecewise, and quadratic) for all major scaling relationships in the study.

**Table S4** Summary statistics of xylem conduit safety margins and safety factors, grouped by xylem type and species.

**Fig. S1** Illustration of the sampling protocol.

**Fig. S2** Graphic outlining the key xylem traits measured in the study.

**Fig. S3** Plot of conduit diameter against distance from stem tip in aboveground dataset, showing how the slope is biased towards points of greater data density.

**Fig. S4** Plot of conduit diameter against external diameter in full dataset, showing how the slope is biased towards points of greater data density.

**Figure S5** Slope and 95% confidence intervals for the relationship between conduit diameter *d* and distance from stem tip *L* as a function of number of *subsampling* and model fitting iterations.

**Figure S6** Slope and 95% confidence intervals for the relationship between conduit diameter *d* and distance from stem tip *L* as a function of number of *bootstrapping* and model fitting iterations.

**Figure S7** Slope and 95% confidence intervals for the relationship between conduit diameter *d* and stem diameter *D_stem_* as a function of number of *subsampling* and model fitting iterations.

**Figure S8** Slope and 95% confidence intervals for the relationship between conduit diameter *d* and stem diameter *D_stem_* as a function of number of *bootstrapping* and model fitting iterations.

**Figure S9** Slope and 95% confidence intervals for the relationship between conduit diameter *d* and root diameter *D_root_* as a function of number of *subsampling* and model fitting iterations.

**Figure S10** Slope and 95% confidence intervals for the relationship between conduit diameter *d* and root diameter *D_root_* as a function of number of *bootstrapping* and model fitting iterations.

**Figure S11** Relationship between hydraulic conduit diameter *d_h_* and distance from stem tip *L* (**A**) and relationship between *d* and *L* fitted using a *subsampled* dataset (**B**).

**Fig. S12** Relationship between conduit diameter *d* and distance from leaf tip *L*, fitted to individual trees in the study.

**Figure S13** Relationship between hydraulic conduit diameter *d_h_* and stem diameter *D_stem_* (**A**) and relationship between *d* and *D_stem_* fitted using a *subsampled* dataset (**B**).

**Figure S14** Relationship between hydraulic conduit diameter *d_h_* and root diameter *D_root_* (**A**) and relationship between *d* and *D_root_* fitted using a *subsampled* dataset (**B**).

**Figure S15** Relationship between stem diameter *D* and distance from stem tip *L* in twigs, branches, and trunk.

**Figure S16** Scatterplot showing the relationship between cell wall thickness *x* and xylem conduit diameter *d* in aboveground organs (i.e., leaves, twigs, branches, and trunk)*.*

**Appendix S1.** Overview of theoretical models predicting the scaling exponents (*α* and *β*) for the relationship between conduit size and path length & organ diameter.

**Note: model variables are described using the original notations. The predicted scaling exponents are reported in the notation used in this article at the beginning of each model description.**

**Pipe model (predicts *α* = 0, *β* = 0)**

The pipe model (Shinozaki *et al.*, 1964a,b) is the first recorded theoretical model in the history of conduit scaling literature that explicitly discusses the dimensions of xylem conduits along the length of stem. In Shinozaki’s pipe model, the quantity of leaves F (ton ha^-1^) existing at a certain height above the ground z (m) is proportional to the quantity of stems C (ton ha∙m^-1^) existing at that same point above the ground, and is given by (Eqn. 2; Shinozaki *et al*. 1964a)

$$F(z)=\chi\times C(z)$$

where χ is a constant (dimensionless). The authors state that

“Analogically speaking, a unit amount of leaves is provided with **a pipe** **whose thickness or cross-sectional area is constant**. The pipe serves both as the vascular passage and as the mechanical support and runs from the leaves to the stem base through all of the intervening strata.” (Shinozaki *et al*. 1964a, pp. 100)

implying that the radius of pipes (i.e., xylem conduits) which deliver sap and nutrients to leaves are invariant with the total distance from the leaves to the stem base. As such, the scaling relationship between pipe or conduit radius *a* (µm) and the length of the pipe from leaves to the base of the stem (or some point along the stem) L (m) is given by

$$a\propto L^{\alpha}$$

where α is the scaling exponent and equals to α = 0. It is noteworthy that Shinozaki’s pipe model was quickly abandoned by plant physiologists as empirical evidence showed that conduits widen with distance from the tip of the plant (Zimmermann, 1978).

**West Brown & Enquist (WBE) model (predicts *α* = ¼ = 0.25, *β* = ⅙ ≈ 0.17)**

The pipe model was followed by the West Brown & Enquist (WBE) model of plant allometry (West *et al.*, 1999) some 35 years after the publication of the former model. WBE model describes the architecture of an allometrically idealized plant using three parameters: *a*, which defines the relationship between the cross-sectional area of the daughter and parent segments; *ā*, which defines the rate at which the conduits inside the segments taper between parent and daughter segments; and *n*, which defines the number of daughter segments branching out from each parent segment, and is assumed to equal to 2 (West *et al.*, 1999). In WBE notation, the parent generation is signified by the letter *k* and the daughter generation is signified as *k*+1. From here, we can define the ratios of daughter and parent segment radii (*r*), conduit radii (*a*), and segment lengths (*l*). The ratio of the daughter segment radius to the parent segment radius is defined as (Box 1; West *et al.*, 1999)

$$\beta_{k}=\frac{r_{k+1}}{r_{k}}=n^{-a/2}$$

The ratio of the daughter conduit radius to the parent conduit radius is similarly defined as (Box 1; West *et al.*, 1999)

$$\bar{\beta}_{k}=\frac{a_{k+1}}{a_{k}}=n^{-\bar{a}/2}$$

Combining equations 3 and 4 yields a scaling relationship between conduit radius and segment radius (Anfodillo *et al.*, 2006)

$$\begin{matrix} a_{k}\propto n^{-\bar{a}/2} \\ r_{k}\propto n^{-a/2} \\ \frac{a_{k}}{r_{k}}\propto\frac{n^{-\bar{a}/2}}{n^{-a/2}} \\ \frac{a_{k}}{r_{k}}\propto1^{\frac{\bar{a}}{a}} \\ a_{k}\propto r_{k}^{\frac{\bar{a}}{a}} \end{matrix}$$

Along with segment radii (Eqn. 1) and conduit radii (Eqn. 2), the ratio of segment lengths (*l*) can be defined as (Box 1; West *et al*., 1999)

$$\gamma_{k}=\frac{l_{k+1}}{l_{k}}=n^{-1/3}$$

For all the terminal segments in a network to be serviced (in this case, the petioles in a plant), the volume of segments must be preserved from one generation to the next. This is broadly known as the “space-filling requirement” of fractal systems, such as a plant network, with *γ_k_* = *n*^-1/3^ being the general property of such systems (West *et al.*, 1997).

Combining equations 3 and 6 yields a scaling relationship between segment radius and length (Anfodillo *et al.*, 2006)

$$\begin{matrix} l_{k}\propto n^{-1/3} \\ r_{k}\propto n^{-a/2} \\ \frac{l_{k}}{r_{k}}\propto\frac{n^{-1/3}}{n^{-a/2}} \\ \frac{l_{k}}{r_{k}}\propto1^{\frac{-1/3}{-a/2}} \\ \frac{l_{k}}{r_{k}}\propto1^{\frac{-1/3}{-1/2a}} \\ \frac{l_{k}}{r_{k}}\propto1^{2/3a} \\ l_{k}\propto r_{k}^{2/3a} \end{matrix}$$

It has been previously demonstrated, both theoretically and empirically (McMahon & Kronauer, 1976; Niklas, 1994), that there exists an optimal allometric relationship between stem lengths and radii

$$l_{k}\propto r_{k}^{A}$$

where *Α* is given by the solution for Greenhill’s buckling formula for slender beams and it equals to 2/3 under the assumption of the elastic similarity model (i.e., that the deflection at the free end of the beam must remain constant relative to the length of the beam; McMahon & Kronauer, 1976; Niklas, 1994). In other words, the radius of any given segment must increase to 2/3^rd^ power with the length of that same segment to resist buckling, i.e., collapse under the weight of the segment. Following from this

$$\begin{matrix} A=\frac{2}{3}a \\ \frac{2}{3}=\frac{2}{3}a \\ a=1 \end{matrix}$$

With *a* = 1, the cross-sectional area of segments is preserved with subsequent generations, which is known as the “area-preserving rule” (or the Da Vinci Rule; West *et al*., 1997). However, it should be noted that consistent 2/3^rd^ power scaling between *l_k_* and *r_k_* is only observed in large branches and tree trunks (West *et al*. 1999). *A* deviates from the 2/3 power prediction towards the tip of the plant, approaching *A* ≈ 2 in peripheral branches and twigs (McMahon & Kronauer, 1976; Bertram, 1989; Fig. S13) as predicted by the flow similarity model (Price *et al*., 2022).

Now we must establish the allometric relationship between conduit radius and segment length. Combining equations 5 and 7 yields

$$\begin{matrix} a_{k}\propto r_{k}^{\frac{\bar{a}}{a}} \\ l_{k}\propto r_{k}^{2/3a} \\ \frac{a_{k}}{l_{k}}\propto\frac{r_{k}^{\frac{\bar{a}}{a}}}{r_{k}^{2/3a}} \\ \frac{a_{k}}{l_{k}}\propto1^{\frac{\frac{\bar{a}}{a}}{2/3a}} \\ a_{k}\propto l_{k}^{\frac{\bar{a}}{2/3}} \end{matrix}$$

When *ā* = 1/6, resistance does not increase with path length (Eqn. 3; West *et al*., 1999), allowing plants to grow taller and deliver an equal supply of water to all the leaves in the canopy. If *ā* < 1/6 (e.g., *ā* = 0 as in Shinozaki’s pipe model; Shinozaki *et al*., 1964a), resistance would quickly increase with path length, greatly limiting the total height that a plant could achieve. Thus

$$\begin{matrix} a_{k}\propto l_{k}^{\frac{1/6}{2/3}} \\ a_{k}\propto l_{k}^{1/4} \end{matrix}$$

meaning that conduit radius for any given segment scales with the length of that segment to the ¼ power.

Now we will define how conduit radius (*a*) scales with path length (*L*). Path length can be calculated by summing the lengths of all the segments from the base of the plant (*l_0_*) to some point along the length of the path (*l_k_*) as (Box 2; West *et al*., 1999)

$$L=\sum_{k=0}^{N} l_{k}=l_{0}+l_{1}+l_{2}+...+l_{N}$$

where *N* is the number of generations. As the length of the parent segments *l_k_* and daughter segments *l_k+1_* scale as shown in Eqn. 6, the total path length (*L*) equals to the sum of individual segment lengths (*l_k_*) at a geometric progression with a rate of *n*^-1/3^ (Box 2; West *et al*., 1999)

$$L=\sum_{k=0}^{N} l_{k}=l_{0}+(l_{1}=l_{0}\times n^{-1/3})+(l_{2}=l_{1}\times n^{-1/3})+...+(l_{N}=l_{N-1}\times n^{-1/3})$$

The sum of the geometric series above, equaling to the total path length *L*, can be expressed in a closed form, which has a general expression

$$S=\frac{a}{(1-r)}$$

where *S* is the sum of the series, *a* is the first term in the series, and *r* is the common ratio. For the WBE plant (Box 2; West *et al*., 1999)

$$L=\frac{l_{0}}{1-n^{-1/3}}$$

and so *L* ∝ *l_0_*, meaning that *L* ∝ *a_0_*, i.e., the conduit radius at the base of the path (e.g., the base of the tree). Further, the equation above is identical to the equation for L provided in Box 2 of West, Brown, and Enquist (1999). Given that *a_k_* ∝ *l_k_*^1/4^ as shown in equation 11,

$$a_{0}\propto l_{0}^{1/4}$$

Re-arranging equation 15 to solve for *l_0_* and substituting it into equation 11 yields

$$\begin{matrix} l_{0}=L\times(1-n^{-1/3}) \\ a_{0}\propto l_{0}^{1/4} \\ a_{0}\propto(L\times(1-n^{-1/3}))^{1/4} \end{matrix}$$

and

$$a_{0}\propto L^{1/4}$$

Thus, following the equations outlined in the WBE model, conduit radius at a particular point along the path length scales with path length (i.e., the distance from the distalmost point of the plant to said point along the path length) to the 1/4^th^ power.

**Height-corrected WBE model (predicts *α* = ⅕ ≈ 0.2, *β* ≈ 0.11)**

It has been noted in the works following West *et al.* (1999) that the equation for *L* (found in Box 2 of West *et al.* (1999), also Eqn. 15 in this document) is an approximation of total path length rather than its true value (Becker *et al.*, 2000; Anfodillo *et al.*, 2006). This is because *L* is ultimately dependent on *N* (i.e., number of segments) in the WBE model, and the closed form of the equation (Eqn. 15) does not account for this dependence. Using a simplified version of Eqn. 3 in Becker *et al.* (2000), Anfodillo *et al.* (2006) define *L* (in Eqn. 11 of their paper) as

$$L=l_{0}\left[(1-n^{-N/3})/(1-n^{-1/3})\right]$$

Indeed, Eqn. 19 yields the same answer as the manual summation of segment lengths (Eqn. 13) regardless of the value of *N*. In practice, equations 15 and 19 yield nearly identical *L* for larger plants (*N* > 10) but can yield drastically different answers for smaller plants (Fig. 5; Anfodillo *et al.* (2006)). As such, Anfodillo *et al.* (2006) recommend using a correction factor that they determined empirically as the slope of the relationship between *L* calculated using Eqn. 15 and *L* calculated using Eqn. 19 (≈0.794). Using this correction factor, Eqn. 18 becomes

$$\begin{matrix} a_{0}\propto L^{0.794(1/4)} \\ a_{0}\propto L^{\sim1/5} \end{matrix}$$

with the scaling exponent for the relationship between conduit radius and path length becoming ~1/5 (0.2). The 1/5^th^ power exponent has also been claimed as the “universal scaling exponent” for the relationship between path length and conduit size by a number of recent papers (Rosell *et al.*, 2017; Olson *et al.*, 2018, 2021).

The relationship between *a_0_* and *L* can also be expressed as the relationship between *a_0_* and *r* using the height-corrected relationship between stem radius, *r* and path length, *L* (see Eqn. 7b in Anfodillo *et al.* 2006). If

$$L\propto r^{0.794A}$$

the relationship between *a_0_* and *r* becomes

$$\begin{matrix} a_{0}\propto L^{0.794\left(\frac{1}{4}\right)} \\ L\propto r^{0.794A} \\ a_{0}\propto r^{0.794A\left[0.794\left(\frac{1}{4}\right)\right]} \\ a_{0}\propto r^{\approx0.11} \end{matrix}$$

**Packed conduit model (predicts *α* = ½ = 0.5, *β* = ⅓ ≈ 0.33)**

The scaling relationship between conduit radius and segment radius predicted by the WBE model (West *et al*., 1999) was challenged by the packed conduit model described in Savage *et al*. (2010). The biggest difference between the former and latter models is that the latter model incorporates branching in the internal (i.e., conduit) network structure of the plant, while the former assumes that branching is exclusive to external (i.e., segment) network and treats the internal network as a series of vertically stacked individual pipes. The scaling exponent of the relationship between conduit and segment radius as predicted by Savage *et al.* (2010) is

$$a_{k}\propto r_{k}^{1/3}$$

Substituting 1/3 for the value of *ā* instead of 1/6 (as predicted by the original WBE model) into Eqn. 11 yields a scaling relationship between conduit radius and segment length as predicted by the packed conduit model

$$\begin{matrix} a_{k}\propto l_{k}^{\frac{1/3}{2/3}} \\ a_{k}\propto l_{k}^{1/2} \end{matrix}$$

and thus the scaling relationship between path length and conduit radius should increase to the ½ power (see also Table 1 in Rossell *et al.* (2017) for identical prediction)

$$\begin{matrix} a_{0}\propto(L\times(1-n^{-1/3}))^{1/2} \\ a_{0}\propto L^{1/2} \end{matrix}$$

**Carbon cost-gain model (predicts *α* = ⅙ ≈ 0.17)**

Following the packed conduit model (Savage *et al*. 2010), Hölttä *et al.* (2011) predicted that the scaling exponent should equal to 0.17 (~1/6) when the net carbon gain (*G_N_*) was maximized by a hypothetical plant (Hölttä *et al.*, 2011). As such, their model uses net carbon gain as the optimization criterion for the rate of conduit widening rather than the minimization of hydraulic resistance and maintenance of conductivity along the vascular network (as in the WBE model). They defined net carbon gain as (Hölttä *et al.*, 2011)

$$G_{N}=G-C_{C}$$

where *G* is the rate of carbon assimilation that a single conduit contributes to over a span of one year under a specific value of stomatal conductance and *C_c_* is the cost of building a single conduit (i.e., the amount of carbon required to construct cell walls surrounding a given conduit). The model plant described in Hölttä *et al.* 2011 follows the structure of the WBE model plant: a series of vertically stacked segments containing pipes which gradually widen towards the base and have leaves only at the tip of the structure. The optimization task of the model is to select a combination of conduit radius *a(i),* the number of conduits *n(i)*, and the leaf water potential *ψ(N)* which maximizes net carbon gain *G_N_* (Eqn. 24). This was done by simultaneously varying five different parameters (*A*, *B*, *C*, *D*, and *E*) as

$$a(i)=Ax^{B}$$

$$n(i)=Cx^{D}$$

$$\psi(N)=E$$

where *x* is the distance from the tip of the model plant. The task of optimizing the model (i.e., finding a combination of the five parameters which maximizes *G_N_*) was performed using a Markov chain Monte Carlo simulation. The full model, including all relevant equations, is described in Hölttä *et al.* 2011 *Theory* section.

**Widened pipe model (predicts *α* = ¼ = 0.25 and ~30% deviation from power-law scaling near base of plant)**

Koçillari *et al.* (2021) predicted that the scaling exponent defining the relationship between xylem conduit diameter and path length equals to α = 0.25, same as the original WBE model, albeit with some important differences. Interestingly, both the original WBE model and the widened pipe model fit a global dataset comprising of plants from over 100 different families reasonably well, while Shinozaki’s pipe model (Shinozaki *et al.*, 1964a) and the packed conduit model (Savage *et al.*, 2010) fit the data poorly (Fig. 2; Koçillari *et al*., 2021).

The widened pipe model is similar to the carbon cost-gain model in that it involves trade-offs between two competing vectors of evolution – maximization of hydraulic conductivity and carbon assimilation and minimization of construction costs of individual conduits. In the context of the widened pipe model, the trade-off is between minimizing hydraulic resistance *R* (i.e., maximizing conductance, *1/R*) and minimizing the rate of conduit widening *W* (Koçillari *et al.*, 2021). The carbon cost-gain model (Hölttä *et al.*, 2011) is more complex than the widened pipe model, however, as it specifically models the changes in the number of conduits along path length, water potential gradients through the plant, the role of pits in hydraulic resistance, etc.

Both models represent an improvement over earlier models which did not explicitly take conduit construction cost into account (Shinozaki *et al.*, 1964a,b; West *et al.*, 1999; Anfodillo *et al.*, 2006; Savage *et al.*, 2010). The widened pipe model uses multiobjective Pareto optimization to calculate the optimal *R-W* trade-off solution (Koçillari *et al.*, 2021). The main result of the model is a closed-form analytical solution for the widening of a single conduit as a function of as a function of path length (or distance from the stem tip of the model plant, *L*), given by

$$\sigma(L)=\sigma_{M}\sqrt{\frac{L}{L_{M}}\left(2-\frac{L}{L_{M}}\right)}\equiv\sigma_{M}F\left(\frac{L}{L_{M}}\right)$$

where *σ(L)* is the cross-sectional area of the conduit at a particular point along the hydraulic path, *L_M_* is the total distance of the path length (from stem tip to base), *σ_M_* is the value of *σ(L)* when *L* = *L_M_*, and *F(x)* is a scaling function which equals to (Koçillari *et al.*, 2021)

$$F(x)=\left[x\left(2-x\right)\right]^{1/2}$$

The rate of conduit widening follows power law behavior near the tip (*σ* ∝ *L*^0.25^), gradually departing from a power law to become nearly invariant with path length near the base of the model plant. The full model is described in the *Materials and Methods* and *Results* section of Koçillari *et al.*, (2021).

**References**

Anfodillo T, Carraro V, Carrer M, Fior C, Rossi S. 2006. Convergent tapering of xylem conduits in different woody species. *New Phytologist* 169: 279–290.

Becker P, Gribben RJ, Lim CM. 2000. Tapered conduits can buffer hydraulic conductance from path-length effects. *Tree Physiology* 20: 965–967.

Bertram JEA. 1989. Size-dependent differential scaling in branches: the mechanical design of trees revisited. *Trees* 3: 241–253.

Hölttä T, Mencuccini M, Nikinmaa E. 2011. A carbon cost-gain model explains the observed patterns of xylem safety and efficiency. *Plant, Cell and Environment* 34: 1819–1834.

Koçillari L, Olson ME, Suweis S, Rocha RP, Lovison A, Cardin F, Dawson TE, Echeverría A, Fajardo A, Lechthaler S, *et al.* 2021. The Widened Pipe Model of plant hydraulic evolution. *Proceedings of the National Academy of Sciences* 118: 1–8.

McMahon TA, Kronauer RE. 1976. Tree structures: Deducing the principle of mechanical design. *Journal of Theoretical Biology* 59: 443–466.

Niklas KJ. 1994. *Plant allometry: the scaling of form and process*. University of Chicago Press.

Olson ME, Anfodillo T, Gleason SM, McCulloh KA. 2021. Tip-to-base xylem conduit widening as an adaptation: causes, consequences, and empirical priorities. *New Phytologist* 229: 1877–1893.

Olson ME, Soriano D, Rosell JA, Anfodillo T, Donoghue MJ, Edwards EJ, León-Gómez C, Dawson T, Julio Camarero Martínez J, Castorena M, *et al.* 2018. Plant height and hydraulic vulnerability to drought and cold. *Proceedings of the National Academy of Sciences of the United States of America* 115: 7551–7556.

Price CA, Drake P, Veneklaas EJ, Renton M. 2022. Flow similarity, stochastic branching, and quarter-power scaling in plants. *Plant Physiology* 190: 1854–1865.

Rosell JA, Olson ME, Anfodillo T. 2017. Scaling of Xylem Vessel Diameter with Plant Size: Causes, Predictions, and Outstanding Questions. *Current Forestry Reports* 3: 46–59.

Savage VM, Bentley LP, Enquist BJ, Sperry JS, Smith DD, Reich PB, Von Allmen EI. 2010. Hydraulic trade-offs and space filling enable better predictions of vascular structure and function in plants. *Proceedings of the National Academy of Sciences of the United States of America* 107: 22722–22727.

Shinozaki K, Yoda K, Hozumi K, Kira T. 1964a. A quantitative analysis of plant form - the pipe model theory: I. Basic analyses. *Japanese Journal of Ecology* 14: 97–105.

Shinozaki K, Yoda K, Hozumi K, Kira T. 1964b. A quantitative analysis of plant form-the pipe model theory: II. Further evidence of the theory and its application in forest ecology. *Japanese Journal of Ecology* 14: 133–139.

West GB, Brown JH, Enquist BJ. 1997. A general model for the origin of allometric scaling laws in biology. *Science* 276: 122–126.

West GB, Brown JH, Enquist BJ. 1999. A general model for the structure and allometry of plant vascular systems. *Nature* 400: 664–667.

Zimmermann MH. 1978. Hydraulic architecture of some diffuse-porous trees. *Canadian Journal of Botany* 56: 2286–2295.

**Appendix S2.** Methods for fitting individual tree data to evaluate within-species variation in scaling exponents.

We used a slightly modified version the bootstrapping method outlined in the *Methods* section of the main text to fit individual tree data. Since the aboveground organs of individual trees were sampled at four distinct locations (leaves, twigs, branch ends, and the base of the trunk), binning the data was impractical, since it would always result in number of bins being equal to or smaller than four. Instead of binning the data and bootstrapping it for each bin, we simply bootstrapped data at each *L*. For each individual, we randomly sampled, with replacement, *n_d,max_* - *n_d,L_* observations at each *L*, where *n_d,max_* is the *L* containing the highest number of raw conduit diameter observations and *n_d,L_* is the number of raw conduit diameter observations at each *L*. The bootstrapped data were then combined with the raw data, yielding a sample size of *n_d,max_* at each *L*. Lastly, as we did not have trunk conduit data for one *T. mertensiana* because the individual was protected and could not be cored due to restrictions by UBC Botanical Garden, we supplemented trunk data with coarse root data for this individual.

**Appendix S3.** Comparing the quality of different statistical model fits for the scaling relationships explored in the study.

Various theories (see Table 1; Appendix S1) predict that the key scaling relationships explored in this study (i.e., *d ∝ L* and *d ∝ D*) follow power law behavior, hence why we treat them as such in the manuscript. However, we also explored different nonlinear fits (i.e., exponential, logarithmic, and piecewise regression) and compared them using the Akaike Information Criterion (AIC). Piecewise regression was also used to determine whether there are any “breakpoints” in the data (e.g., points where the linear trend for the relationship between *d* and *L* or *D* suddenly changes to follow a different trend). Power law, logarithmic, and exponential models were fitted using linear regression via the *lm* function in R. For power law and piecewise regression models, both the X and Y were log-transformed prior to fitting; for the logarithmic model, only the X variable was log-transformed; and for the exponential model, only the Y variable was log-transformed.

**Table S1.** Summary of xylem conduit traits measured in the study. *n* (ind.): number of individuals in study; *n* (conduits): number of conduits analysed for each species; *H*: total plant height from stem tip to base (in m); ll: leaf length (in mm); *d_x̄_*: mean conduit diameter; *d_h_*: mean hydraulic conduit diameter; *x*_All_: cell wall thickness across the growth rings; *x*_PX_: cell wall thickness in primary xylem only (i.e., in leaves, twigs, and very fine roots); *x*_EW_: cell wall thickness in earlywood only; *x*_LW_: cell wall thickness in latewood only; *(t/b)^2^_All_*: double wall thickness to cell wall span ratio across the growth rings; *(t/b)^2^_PX_*: double wall thickness to cell wall span ratio in primary xylem only (i.e., in leaves, twigs, and very fine roots); *(t/b)^2^_EW_*: double wall thickness to cell wall span ratio in earlywood only; *(t/b)^2^_LW_*: double wall thickness to cell wall span ratio in latewood only.

|  | ***Callitropsis nootkatensis*** | ***Picea sitchensis*** | ***Tsuga heterophylla*** | ***Tsuga mertensiana*** | ***Thuja plicata*** | Mean |
| --- | --- | --- | --- | --- | --- | --- |
| ***n* (ind.)** | 5 | 5 | 6 | 5 | 6 |  |
| ***n* (conduits)** | 120,249 | 69,392 | 142,499 | 110,728 | 137,181 |  |
| ***H* (m)** | 13.11 (11.87-14.17) | 15.33 (14.26-17.06) | 12.11 (2.87-23.87) | 8.96 (5.91-11.51) | 13.96 (3.09-28.26) | 12.69 |
| **ll (mm)** | 3.98 (3.55-4.4) | 15.31 (12.24-17.97) | 16.1 (14-19.09) | 14.56 (12.69-16.22) | 3.35 (3.09-3.68) | 10.59 |
| ***d_x̄_* (µm)** | 9.5 (3.46-20.18) | 13.76 (5.03-27.63) | 9.56 (4.17-21.66) | 9.95 (4.66-23.03) | 10.52 (3.69-24.79) | 10.66 |
| ***d_h_* (µm)** | 12.62 (4.22-27.93) | 20.3 (5.59-45.02) | 13.84 (4.83-32.61) | 13.87 (5.86-32.84) | 14.48 (4.22-32.19) | 15.02 |
| ***x*_All_ (µm)** | 3.13 (1.78-5.19) | 3.64 (1.64-5.85) | 3.41 (1.38-5.83) | 3.33 (1.51-5.34) | 3.16 (1.75-4.59) | 3.33 |
| ***x*_PX_ (µm)** | 2.3 (1.78-2.85) | 2.28 (1.64-3.25) | 2.31 (1.38-4.29) | 2.23 (1.51-3.56) | 2.55 (1.75-3.9) | 2.33 |
| ***x*_EW_ (µm)** | 3.31 (2.38-4.15) | 3.93 (1.64-5.31) | 3.49 (2.5-5.07) | 3.53 (2.73-4.96) | 3.02 (2.02-4.24) | 3.46 |
| ***x*_LW_ (µm)** | 3.94 (2.77-5.19) | 5.06 (1.86-6.43) | 4.44 (2.9-6.02) | 4.54 (3.62-5.48) | 4.14 (2.57-5.82) | 4.42 |
| ***(t/b)^2^_All_*** | 0.63 (0.16-4.3) | 0.79 (0.08-5.34) | 1.02 (0.12-7.19) | 0.63 (0.11-1.79) | 0.64 (0.1-2.54) | 0.74 |
| ***(t/b)^2^_PX_*** | 0.56 (0.17-1.1) | 0.43 (0.08-1.34) | 0.44 (0.16-0.95) | 0.38 (0.11-0.82) | 0.58 (0.26-0.97) | 0.48 |
| ***(t/b)^2^_EW_*** | 0.15 (0.08-0.32) | 0.1 (0.06-0.18) | 0.15 (0.05-0.31) | 0.15 (0.06-0.22) | 0.11 (0.04-0.23) | 0.13 |
| ***(t/b)^2^_LW_*** | 0.84 (0.27-4.31) | 1.43 (0.15-5.48) | 1.6 (0.22-7.19) | 1.05 (0.3-2.68) | 0.98 (0.17-2.64) | 1.18 |

**Table S2.** Slopes and 95% CIs of key scaling relationships in the study, obtained by fitting the data using both standardized major axis (SMA) and ordinary least squares (OLS) regression.

| **X variable** | **Y variable** | **SMA slope**  **(95% CI)** | **OLS slope**  **(95% CI)** | **Figure** |
| --- | --- | --- | --- | --- |
| *L* | *d* (bootstrapped) | 0.23 (0.23 - 0.23) | 0.12 (0.12 - 0.12) | 2A |
| *L* | *d* (subsampled) | 0.23 (0.22 - 0.23) | 0.11 (0.11 - 0.12) | S12B |
| *L* | *d_h_* | 0.20 (0.18 - 0.22) | 0.18 (0.16 - 0.20) | S12A |
| *D_stem_* | *d* (bootstrapped) | 0.32 (0.32 - 0.32) | 0.22 (0.22 - 0.22) | 2B |
| *D_stem_* | *d* (subsampled) | 0.32 (0.31 - 0.32) | 0.22 (0.22 - 0.22) | S13B |
| *D_stem_* | *d_h_* | 0.28 (0.25 - 0.31) | 0.25 (0.23 - 0.28) | S13A |
| *D_root_* | *d* (bootstrapped) | 0.42 (0.42 - 0.42) | 0.18 (0.18 - 0.18) | 2C |
| *D_root_* | *d* (subsampled) | 0.42 (0.40 - 0.44) | 0.19 (0.17 - 0.21) | S14B |
| *D_root_* | *d_h_* | 0.33 (0.29 - 0.38) | 0.25 (0.21 - 0.30) | S14A |

**Table S3.** Comparisons of different model fits (power law, logarithmic, exponential, piecewise, and quadratic) for all major scaling relationships in the study (see Appendix S3 for methods). Model fits were compared using the Akaike information criterion (AIC) and the model that best fit the data (i.e., had the lowest AIC value) was bolded.

| **Dependent variable** | **Independent variable** | **Figure** | **Model fit** | **Model AIC** | **Estimated value of independent variable at breakpoint (piecewise only)** |
| --- | --- | --- | --- | --- | --- |
| *d* | *L* | 2A | Power law | -424,030 |  |
| *d* | *L* | 2A | Logarithmic | 9,118,274 |  |
| *d* | *L* | 2A | Exponential | -551,785 |  |
| ***d*** | ***L*** | **2A** | **Piecewise** | **-602,548** | **1.03 m** |
| *d* | *L* | 2A | Quadratic | -595,604 |  |
| *d* | *D_stem_* | 2B | Power law | -402,388 |  |
| *d* | *D_stem_* | 2B | Logarithmic | -157,418 |  |
| *d* | *D_stem_* | 2B | Exponential | -199,659 |  |
| ***d*** | ***D_stem_*** | **2B** | **Piecewise** | **-409,470** | **0.14 m** |
| *d* | *D_stem_* | 2B | Quadratic | -404,950 |  |
| *d* | *D_root_* | 2C | Power law | -162,991 |  |
| *d* | *D_root_* | 2C | Logarithmic | 5,518,587 |  |
| *d* | *D_root_* | 2C | Exponential | -46,291 |  |
| ***d*** | ***D_root_*** | **2C** | **Piecewise** | **-241,445** | **0.008 m** |
| *d* | *D_root_* | 2C | Quadratic | -227,684 |  |

**Table S4.** Summary statistics of xylem conduit safety factors, grouped by xylem type (primary, secondary earlywood, and secondary latewood xylem) and species.

|  | **Xylem** | ***Callitropsis nootkatensis*** | ***Picea sitchensis*** | ***Tsuga heterophylla*** | ***Tsuga mertensiana*** | ***Thuja plicata*** | **All species** |
| --- | --- | --- | --- | --- | --- | --- | --- |
| **Safety factor, median** | Primary xylem | 22.36 | 12.70 | 16.67 | 10.98 | 15.98 | 15.68 |
| **Safety factor, 5th percentile** | Primary xylem | 8.18 | 4.35 | 4.57 | 3.65 | 5.12 | 4.70 |
| **Safety factor, 95th percentile** | Primary xylem | 101.49 | 54.22 | 64.36 | 39.19 | 70.92 | 70.65 |
| **Safety factor, IQR** | Primary xylem | 13.56 - 37.64 | 7.72 - 22.31 | 9.76 - 29.27 | 6.69 - 18.72 | 9.65 - 26.74 | 9.38 - 27.87 |
| **Safety factor, median** | Earlywood | 11.25 | 6.89 | 12.13 | 15.51 | 8.23 | 10.01 |
| **Safety factor, 5th percentile** | Earlywood | 4.79 | 1.98 | 5.11 | 7.30 | 2.24 | 2.81 |
| **Safety factor, 95th percentile** | Earlywood | 23.56 | 19.91 | 26.43 | 28.86 | 20.07 | 23.33 |
| **Safety factor, IQR** | Earlywood | 8.13 - 15.4 | 4.07 - 11.22 | 8.66 - 16.67 | 11.61 - 20.17 | 5 - 12.14 | 6.43 - 14.46 |
| **Safety factor, median** | Latewood | 39.60 | 78.08 | 106.46 | 53.23 | 60.69 | 62.21 |
| **Safety factor, 5th percentile** | Latewood | 11.11 | 11.34 | 18.78 | 18.03 | 12.30 | 13.80 |
| **Safety factor, 95th percentile** | Latewood | 448.88 | 978.51 | 889.48 | 315.17 | 453.86 | 620.21 |
| **Safety factor, IQR** | Latewood | 39.59 - 11.1 | 78.07 - 11.33 | 106.46 - 18.78 | 53.23 - 18.02 | 60.68 - 12.3 | 30.08 - 160.61 |


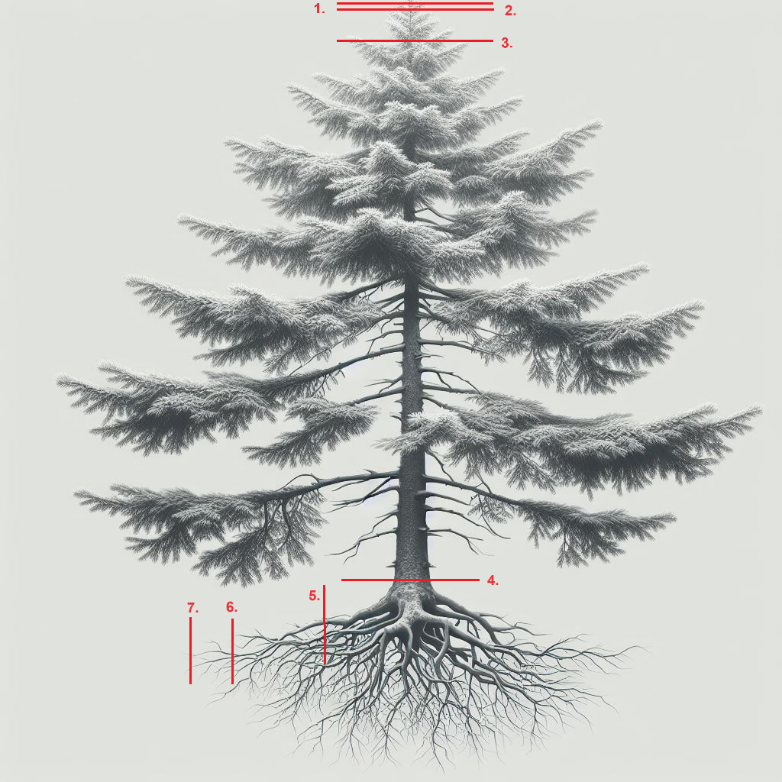


**Figure S1.** Illustration of the sampling protocol. Each individual (*N* = 27) was sampled at seven points along the hydraulic pathway corresponding to distinct organs: 1) leaf; 2) twig; 3) base of the terminal branch; 4) base of the trunk above the root collar; 5) coarse root; 6) fine root; 7) very fine root. Image of the tree silhouette was generated using DALL-E 3.

**
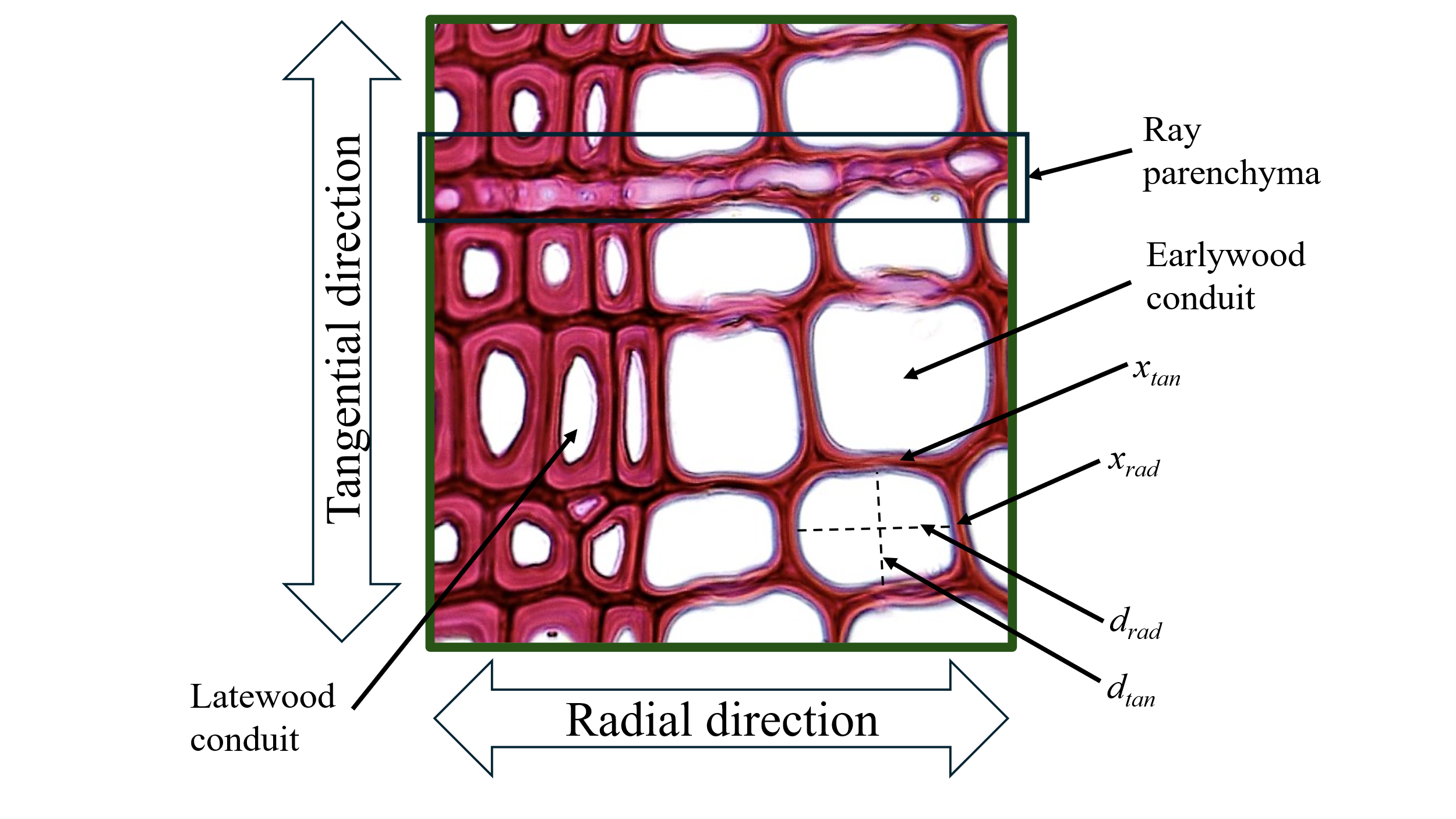
**

**Figure S2.** Graphic outlining the key xylem traits measured in the study. Xylem conduit diameter (*d*) and cell wall thickness (*x*) were measured in radial direction (from centre of vascular bundle toward the epidermis in leaves, twigs, and fine roots, and from bark to pith in woody samples, following the ray parenchyma – see graphic) and tangential direction (perpendicular to the radial direction). Xylem conduit diameter (*d*) is the diameter of the lumen (hollow central space) and was calculated by taking the average of the radial (*d_rad_*) and tangential (*d_tan_*) measurements. Each conduit is bordered by a cell wall of a specific thickness (*x*) in both the radial (*x_rad_*) and tangential direction *x_tan_*). Measurements of *x* are integrated over the length of the cell wall, from the thinnest central portion (where the arrows are pointing to) to the corner of the lumen (see Prendin *et al*. 2017 for details on how cell wall thickness is measured). For all measured conduits, thickness-to-span ratio (*t/b^2^*) was calculated using both radial (*2x_rad_* / *d_rad_*)^2^ and tangential measurements (*2x_tan_* / *d_tan_*)^2^ of cell wall thickness and conduit diameter, and the smaller ratio was selected (reflecting the cell wall more likely to collapse, see Hacke *et al*. 2001). Mork’s index was used to demarcate between earlywood (*4x_tan_* / *d_rad_* < 1) and latewood conduits (*4x_tan_* / *d_rad_* ≥ 1) – the graphic shows an example of an earlywood and latewood conduit.


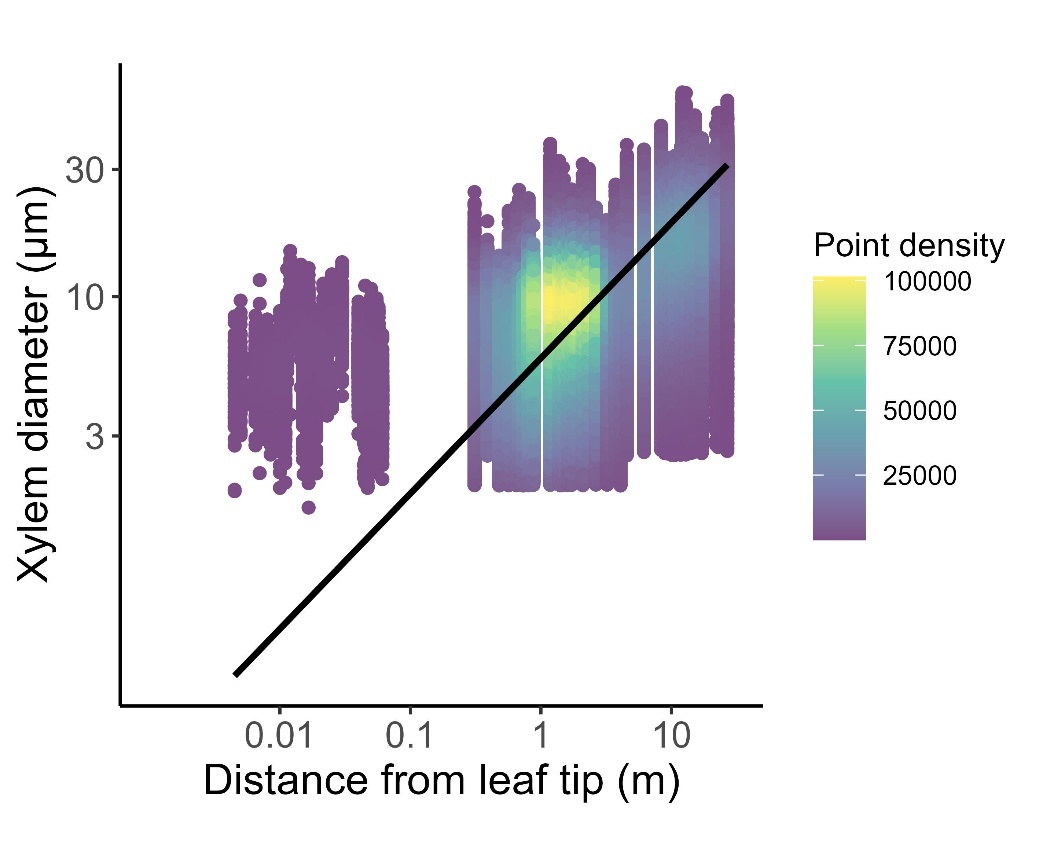


**Figure S3.** Relationship between conduit diameter *d* and distance from leaf tip *L* yields *α* = 0.5 (95% CI = 0.5 – 0.51, *r^2^* = 0.23, *p* < 2.22 ˣ 10^-16^, *n* = 441,904) when fitted using raw (non-bootstrapped) data. Note that the distribution of conduit diameters *d* across path length *L* is highly uneven, with *L* > 1 having a much greater frequency of conduits than *L* < 1. This uneven distribution biases the model fit (including the slope) towards areas with greater data density.


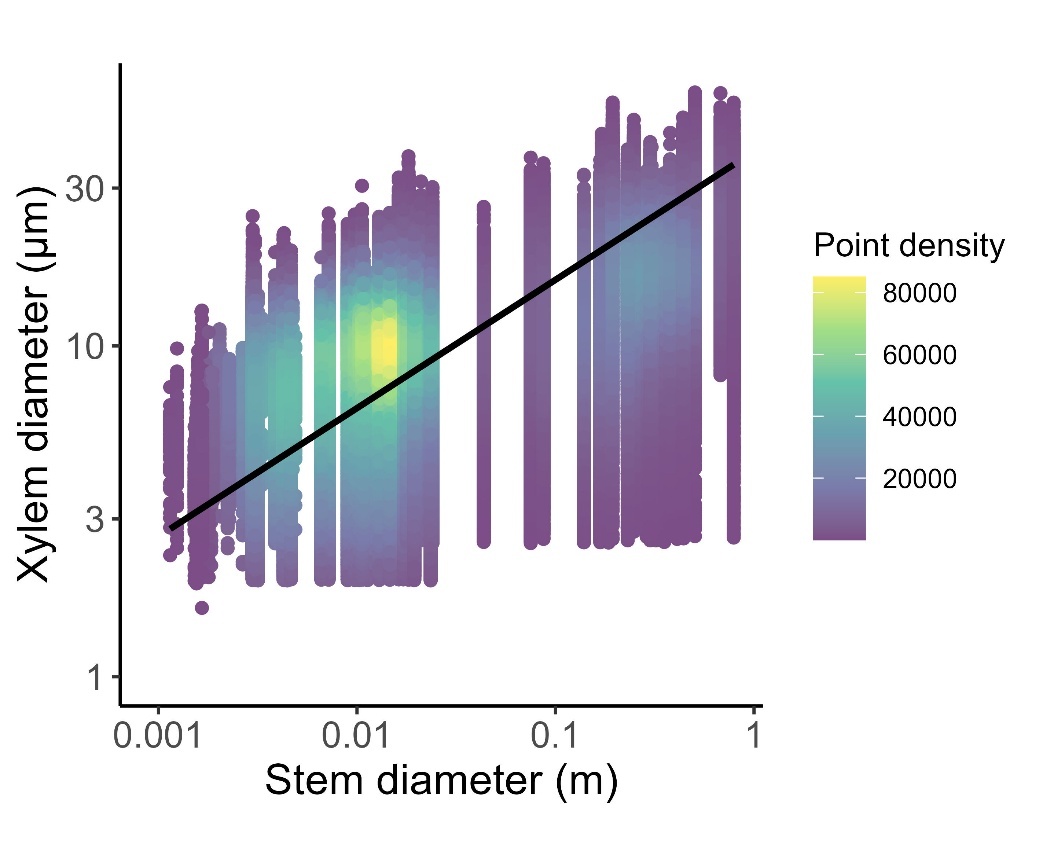


**Figure S4.** Relationship between conduit diameter *d* and external stem diameter *D_stem_* yields *β* = 0.39, (95% CI = 0.39 – 0.39, *r^2^* = 0.29, *p* < 2.22 ˣ 10^-16^, *n* = 441,015) when fitted using raw (non-bootstrapped) data. Note that the distribution of conduits *d* across organ diameters *D_stem_* is uneven, with *D_stem_* ~ 0.01 m having a much greater frequency of conduits than lesser or greater values of *D_stem_*. This uneven distribution biases the model fit (including the slope) towards areas with greater data density.


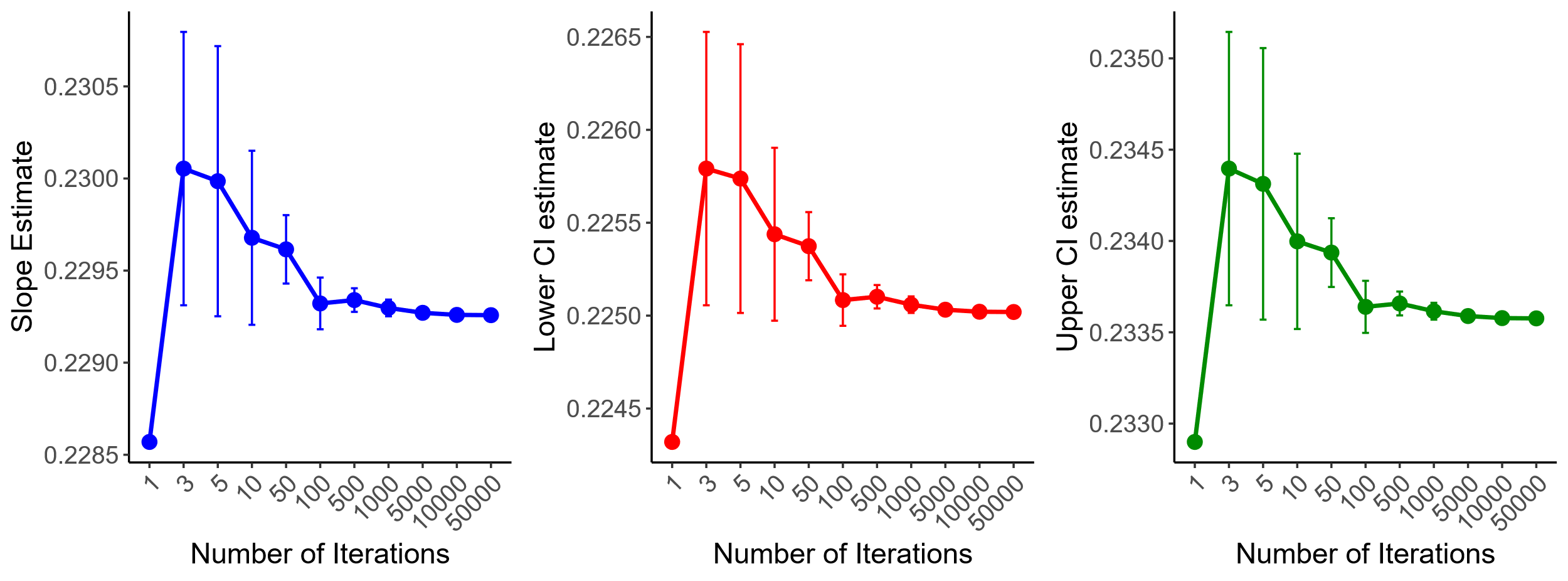


**Figure S5.** Slope and 95% confidence intervals for the relationship between conduit diameter (*d*) and distance from leaf tip (*L*) using the subsampling approach. First, the raw dataset was split into equally spaced logarithmic bins containing a minimum of *n* = 100 observations in each bin. *L* was used as the binning variable. The frequency of observations in each bin was then calculated, and bin with the lowest number of raw conduit diameter observations *(n_d,min_)* was identified. We then sampled, without replacement, *n_d,min_* observations from each bin. The dataset containing the sampled *d* and *L* values was fitted with a standard major axis regression model using the smatr package in R. The resampling and model fitting procedure was repeated for a preset number of iterations (1,3,5,10,50,100,500,1000,10000,50000) to determine the point at which the slope and confidence intervals converge (i.e., no longer vary with increasing number of iterations).


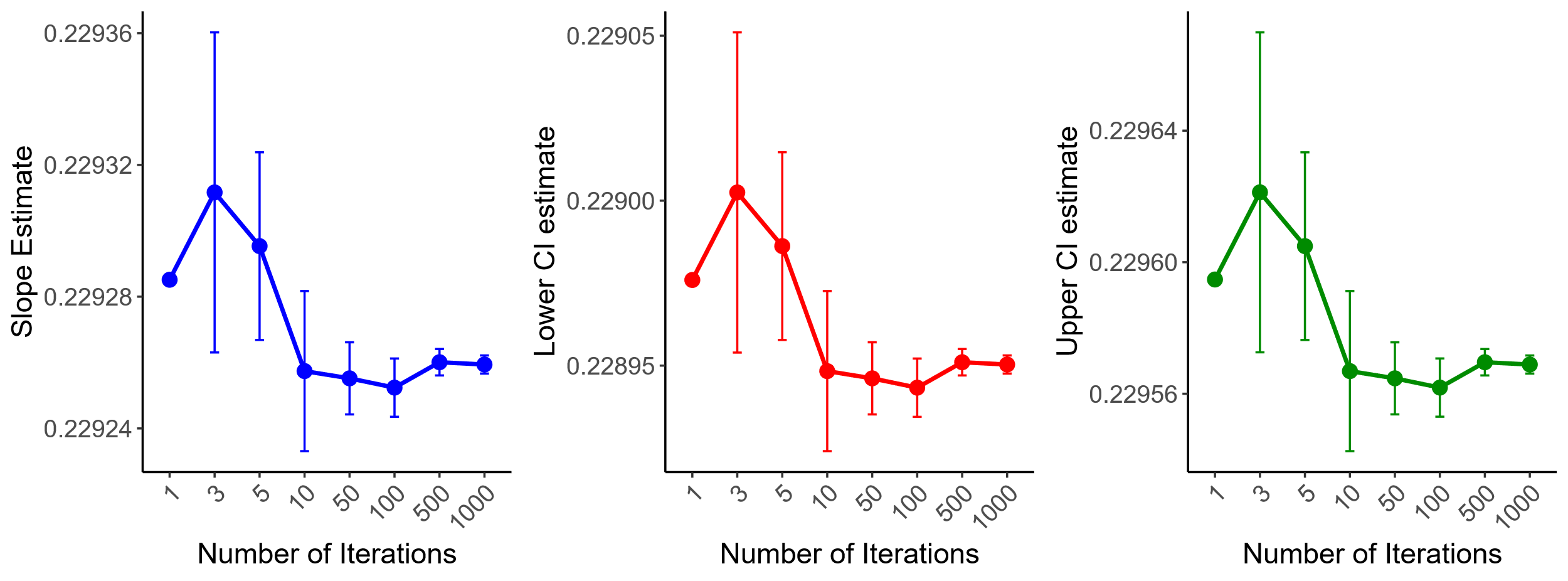


**Figure S6.** Slope and 95% confidence intervals for the relationship between conduit diameter (*d*) and distance from leaf tip (*L*) using the bootstrapping approach. First, the raw dataset was split into equally spaced logarithmic bins containing a minimum of *n* = 100 observations in each bin. *L* was used as the binning variable. The frequency of observations in each bin was then calculated, and bin with the highest number of raw conduit diameter observations *(n_d,max_)* was identified. We then randomly sampled, with replacement, *n_d,max_ -* *n_d,bin_* observations from each of the bin, where *n_d,bin_* is the number of raw conduit diameter observations in each bin. The bootstrapped data was then combined with raw data, yielding a sample size of *n_d,max_* in each bin. The dataset containing the sampled *d* and *L* values was fitted with a standard major axis regression model using the smatr package in R. The resampling and model fitting procedure was repeated for a preset number of iterations (1,3,5,10,50,100,500,1000) to determine the point at which the slope and confidence intervals converge (i.e., no longer vary with increasing number of iterations).


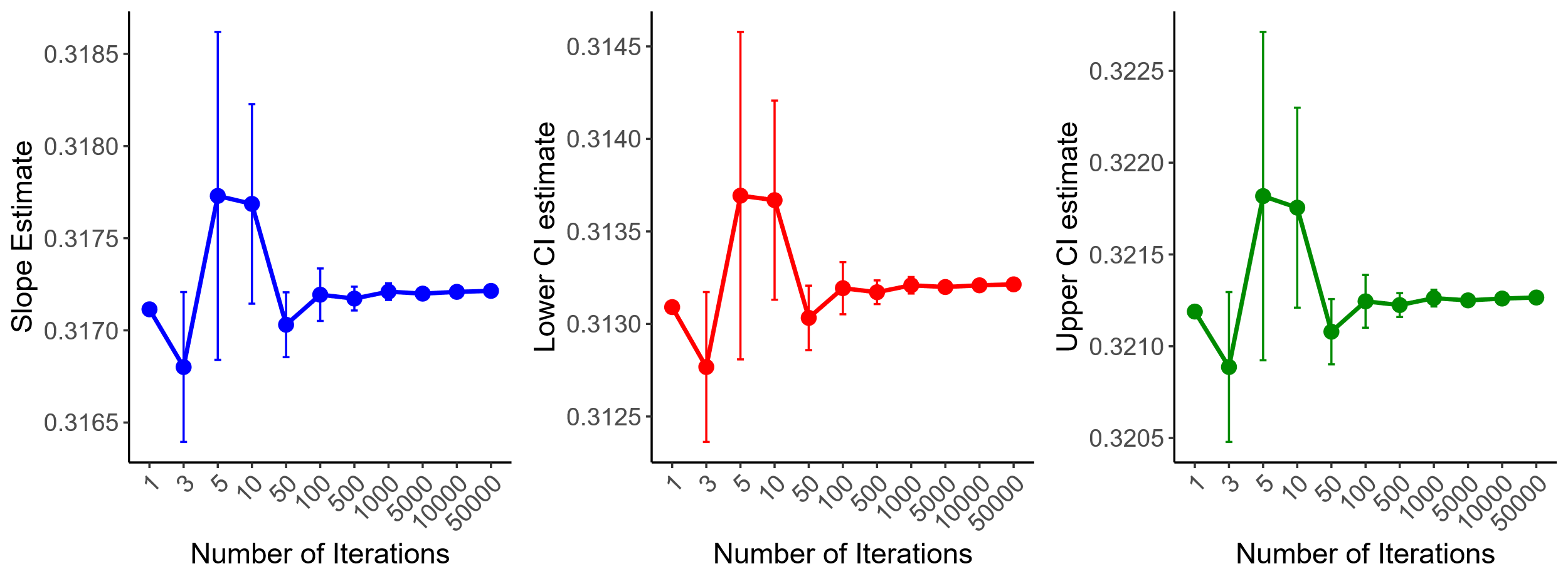


**Figure S7.** Slope and 95% confidence intervals for the relationship between conduit diameter (*d*) and stem diameter (*D_stem_*) using the *subsampling* approach. First, the raw dataset was split into equally spaced logarithmic bins containing a minimum of *n* = 100 observations in each bin. *D_stem_* was used as the binning variable. The frequency of observations in each bin was then calculated, and bin with the lowest number of raw conduit diameter observations *(n_d,min_)* was identified. We then sampled, without replacement, *n_d,min_* observations from each bin. The dataset containing the sampled *d* and *D_stem_* values was fitted with a standard major axis regression model using the *smatr* package in R. The resampling and model fitting procedure was repeated for a preset number of iterations (1,3,5,10,50,100,500,1000,10000,50000) to determine the point at which the slope and confidence intervals converge (i.e., no longer vary with increasing number of iterations).


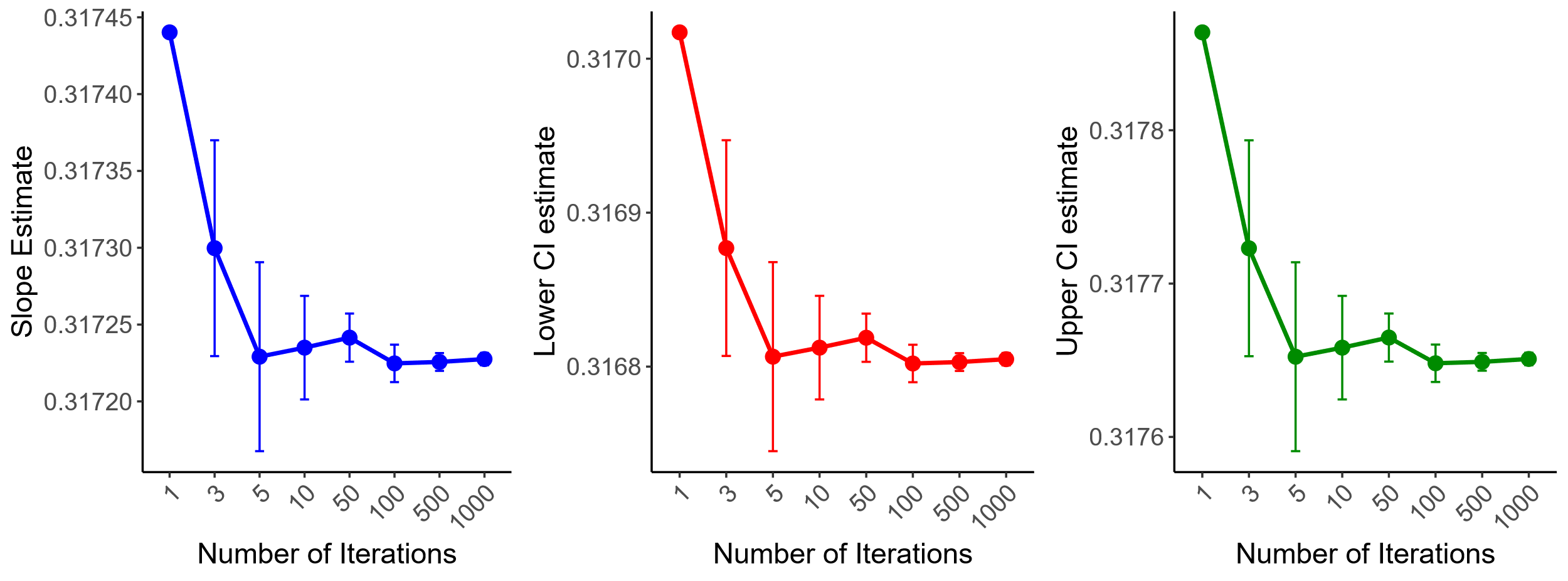


**Figure S8.** Slope and 95% confidence intervals for the relationship between conduit diameter (*d*) and stem diameter (*D_stem_*) using the *bootstrapping* approach. First, the raw dataset was split into equally spaced logarithmic bins containing a minimum of *n* = 100 observations in each bin. *D_stem_* was used as the binning variable. The frequency of observations in each bin was then calculated, and bin with the highest number of raw conduit diameter observations *(n_d,max_)* was identified. We then randomly sampled, with replacement, *n_d,max_ -* *n_d,bin_* observations from each bin, where *n_d,bin_* is the number of raw conduit diameter observations in each bin. The bootstrapped data was then combined with raw data, yielding a sample size of *n_d,max_* in each bin. The dataset containing the sampled *d* and *D_stem_* values was fitted with a standard major axis regression model using the *smatr* package in R. The resampling and model fitting procedure was repeated for a preset number of iterations (1,3,5,10,50,100,500,1000) to determine the point at which the slope and confidence intervals converge (i.e., no longer vary with increasing number of iterations).

**
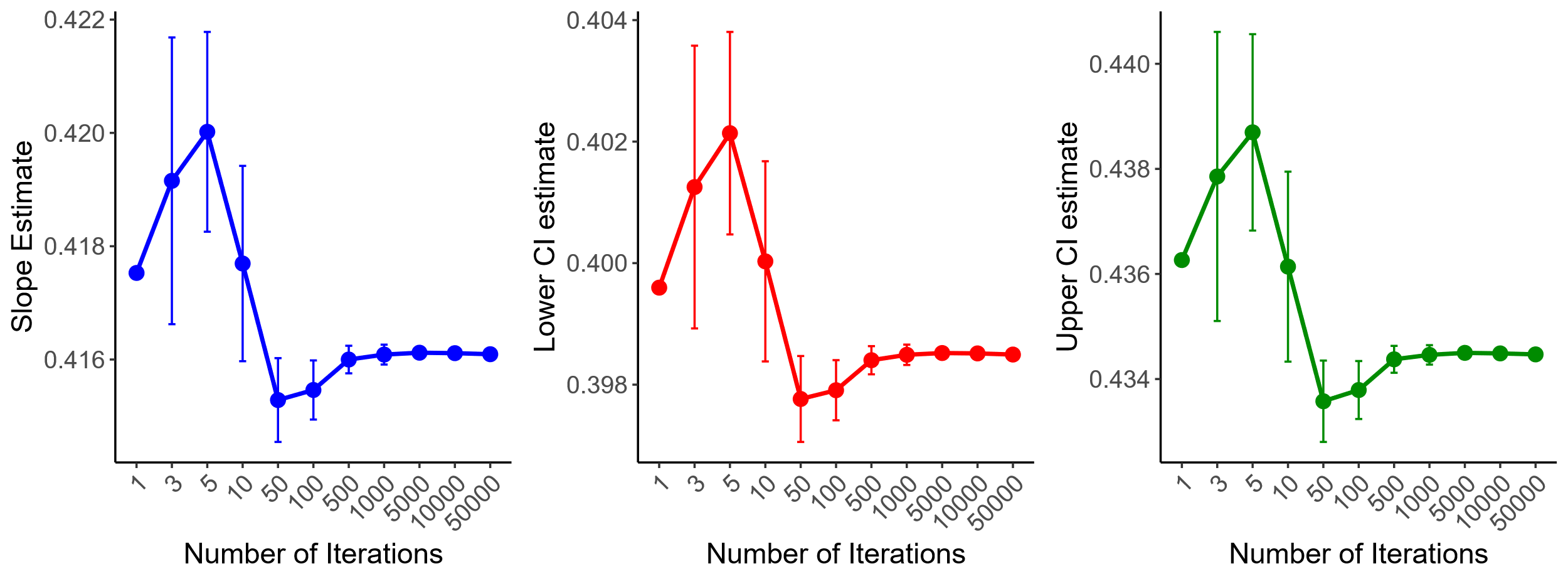
**

**Figure S9.** Slope and 95% confidence intervals for the relationship between conduit diameter (*d*) and root diameter (*D_root_*) using the *subsampling* approach. First, the raw dataset was split into equally spaced logarithmic bins containing a minimum of *n* = 100 observations in each bin. *D_root_* was used as the binning variable. The frequency of observations in each bin was then calculated, and bin with the lowest number of raw conduit diameter observations *(n_d,min_)* was identified. We then sampled, without replacement, *n_d,min_* observations from each bin. The dataset containing the sampled *d* and *D_root_* values was fitted with a standard major axis regression model using the *smatr* package in R. The resampling and model fitting procedure was repeated for a preset number of iterations (1,3,5,10,50,100,500,1000,10000,50000) to determine the point at which the slope and confidence intervals converge (i.e., no longer vary with increasing number of iterations).


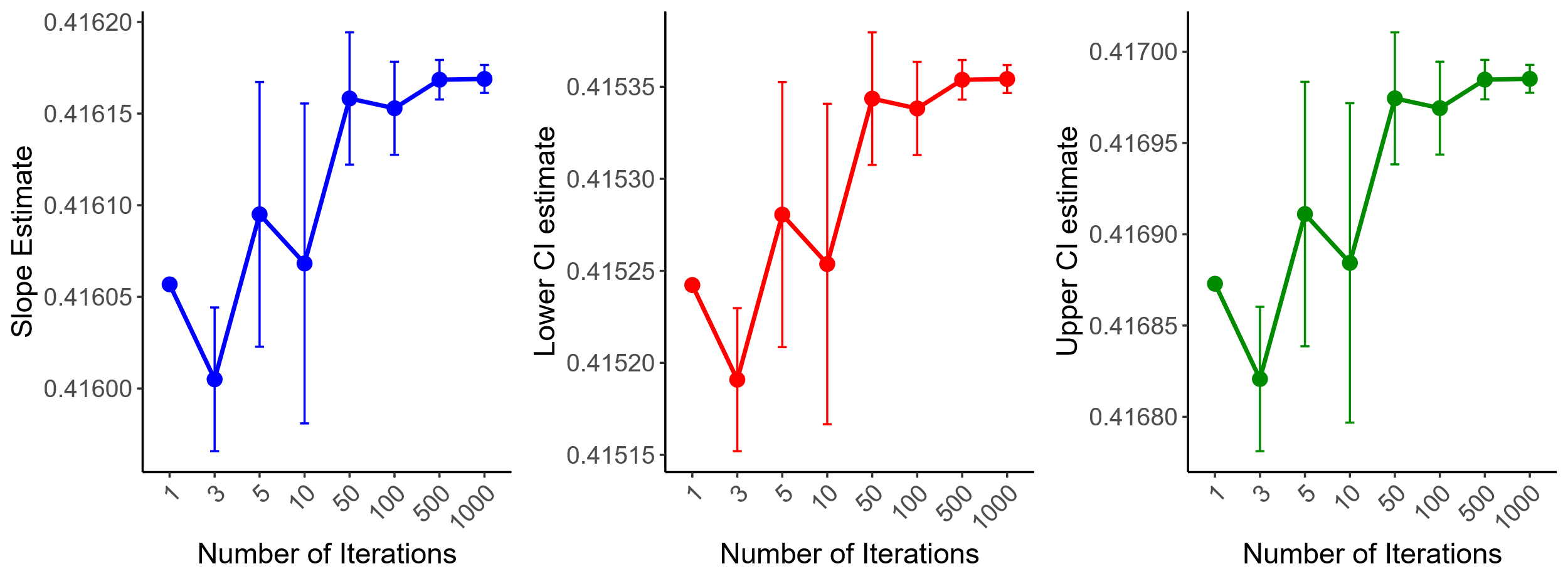


**Figure S10.** Slope and 95% confidence intervals for the relationship between conduit diameter (*d*) and root diameter (*D_root_*) using the *bootstrapping* approach. First, the raw dataset was split into equally spaced logarithmic bins containing a minimum of *n* = 100 observations in each bin. *D_root_* was used as the binning variable. The frequency of observations in each bin was then calculated, and bin with the highest number of raw conduit diameter observations *(n_d,max_)* was identified. We then randomly sampled, with replacement, *n_d,max_ -* *n_d,bin_* observations from each bin, where *n_d,bin_* is the number of raw conduit diameter observations in each bin. The bootstrapped data was then combined with raw data, yielding a sample size of *n_d,max_* in each bin. The dataset containing the sampled *d* and *D_root_* values was fitted with a standard major axis regression model using the *smatr* package in R. The resampling and model fitting procedure was repeated for a preset number of iterations (1,3,5,10,50,100,500,1000) to determine the point at which the slope and confidence intervals converge (i.e., no longer vary with increasing number of iterations).


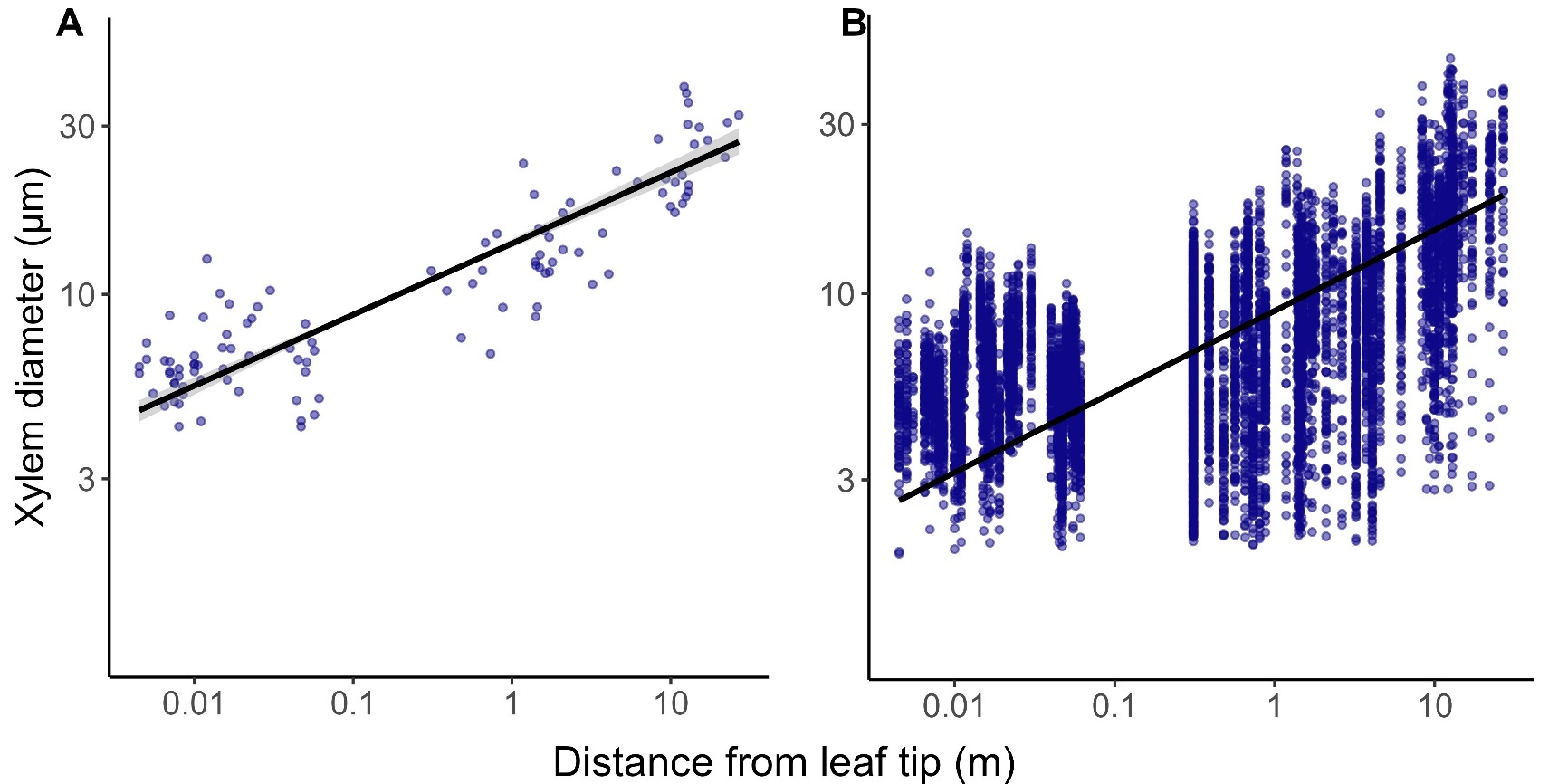


**Figure S11. (A)** Relationship between hydraulic conduit diameter *d_h_* (i.e., mean hydraulically-weighted conduit diameter; Eqn. 7) and distance from leaf tip *L* (i.e., path length; *α* = 0.20, 95% confidence interval (CI) = 0.18 – 0.22, *p* = 2.22 × 10^-16^, *r^2^* = 0.79) and **(B)** relationship between conduit diameter *d* and *L* fitted using a *subsampled* dataset (*α =* 0.23, 95% CI: 0.22 – 0.23, *p*=2.22 × 10^-16^, *r^2^*=0.25).


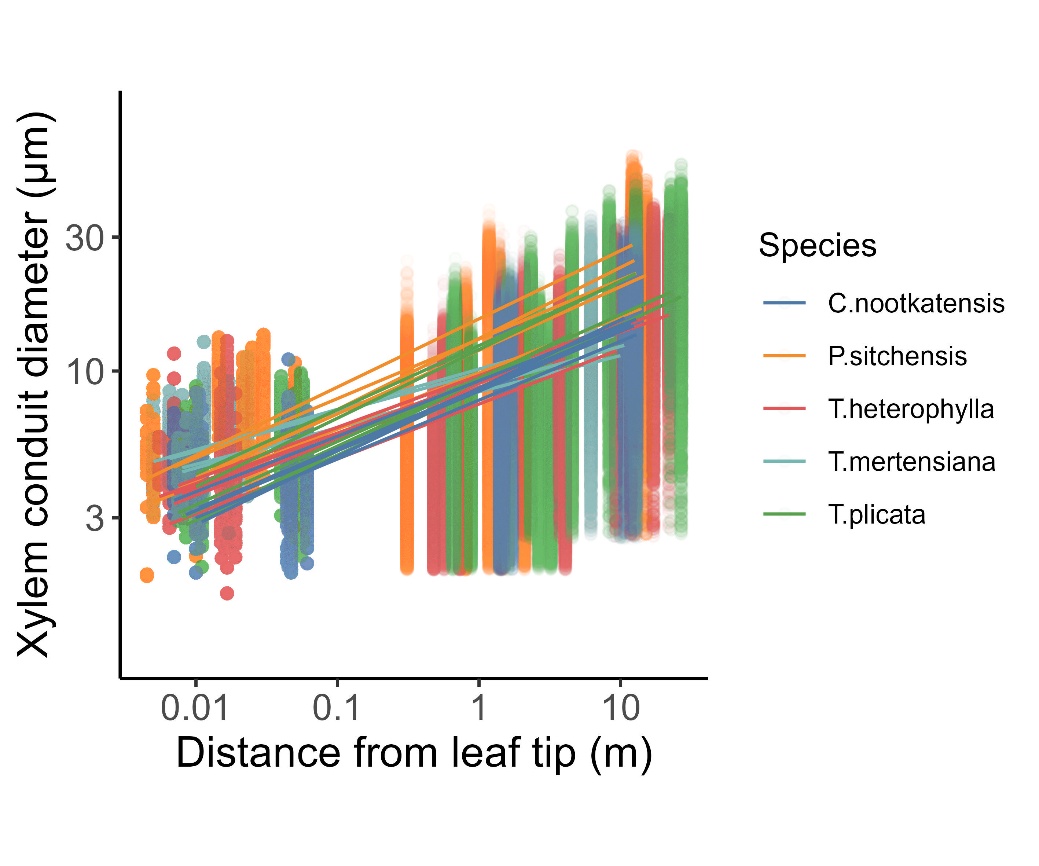


**Figure S12.** Relationship between conduit diameter *d* and distance from leaf tip *L*, fitted to individual trees in the study. We used a modified bootstrapping method for fitting individual tree data (see Appendix S2). When slopes were averaged across all individuals, the mean path-length scaling, *α,* equaled to 0.20 (95% CI: 0.19 – 0.22). Slopes also varied significantly (*p* < 2.22 ˣ 10^-16^) between species: *α* = 0.22 (CI: 0.22 – 0.22) for *C. nootkatensis*; *α* = 0.23 (CI: 0.23 – 0.23) for *P. sitchensis*; *α* = 0.19 (CI: 0.19 – 0.19) for *T. heterophylla*; *α* = 0.14 (CI: 0.14 – 0.14) for *T. mertensiana*; and *α* = 0.24 (0.24 – 0.24) for *T. plicata*.


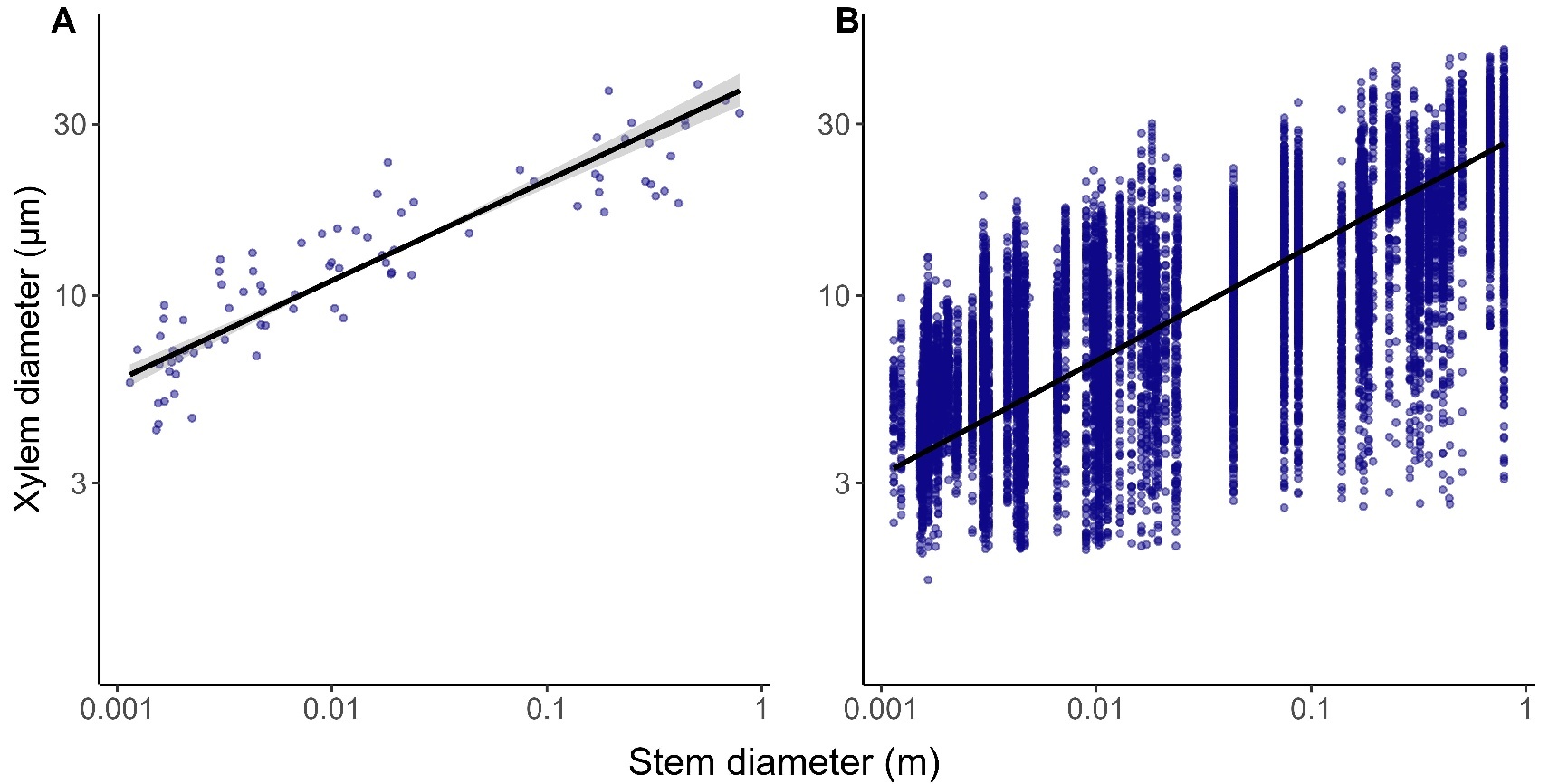


**Figure S13. (A)** Relationship between hydraulic conduit diameter *d_h_* (i.e., mean hydraulically-weighted conduit diameter; Eqn. 7) and external stem diameter *D_stem_* (*β* = 0.28, 95% confidence interval (CI) = 0.25 – 0.31, *p* = 2.22 × 10^-16^, *r^2^* = 0.82) and **(B)** relationship between conduit diameter and external stem diameter fitted using a *subsampled* dataset (*β =* 0.32, 95% CI: 0.31 – 0.32, *p*=2.22 × 10^-16^, *r^2^*=0.48).


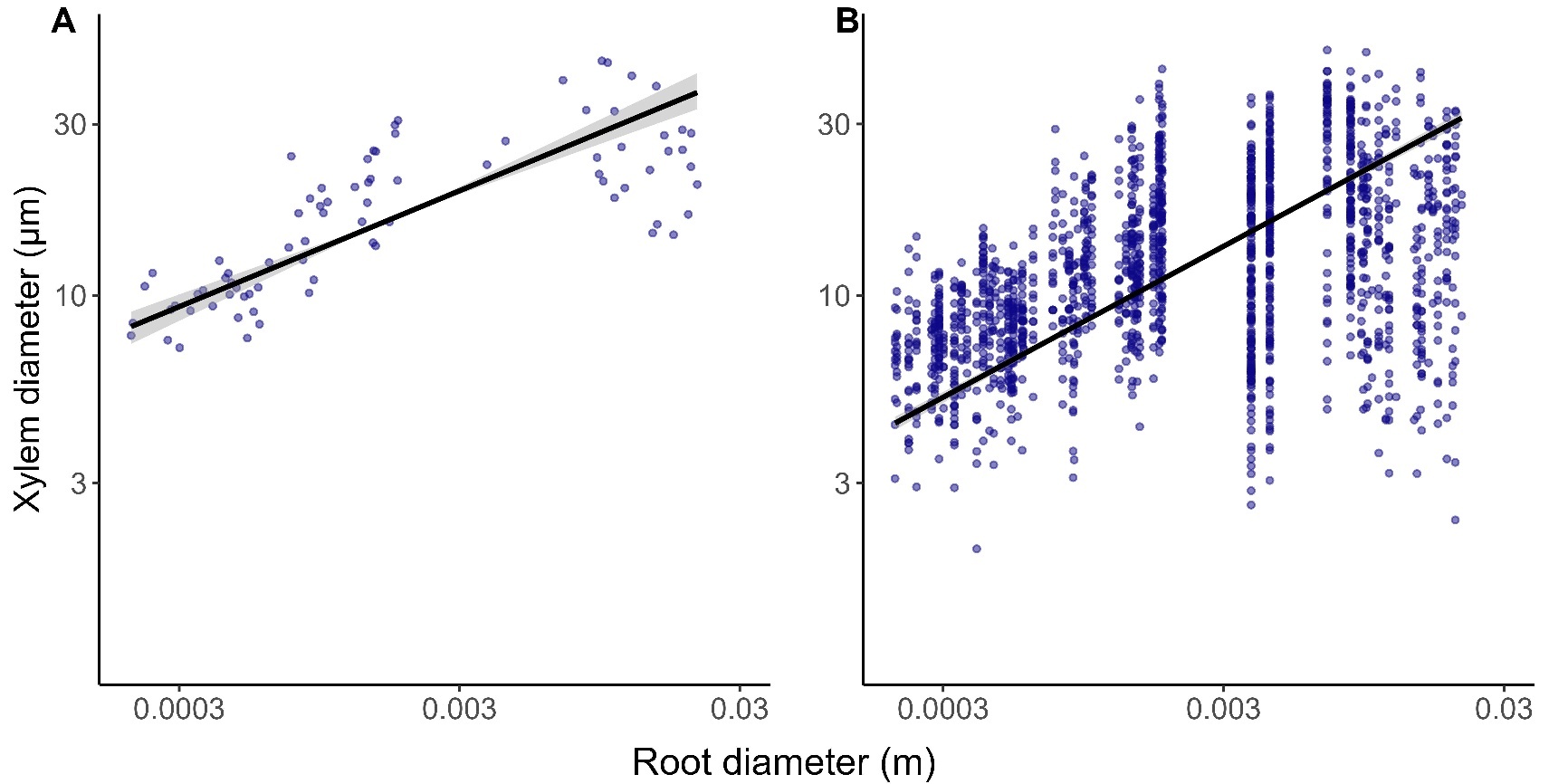


**Figure S14. (A)** Relationship between hydraulic conduit diameter *d_h_* (i.e., mean hydraulically-weighted conduit diameter; Eqn. 7) and external root diameter *D_root_* (*β* = 0.33, 95% confidence interval (CI) = 0.29 – 0.38, *p* = 2.22 × 10^-16^, *r^2^* = 0.59) and **(B)** relationship between conduit diameter and external root diameter fitted using a *subsampled* dataset (*β =* 0.42, 95% CI: 0.40 – 0.44, *p* = 2.22 × 10^-16^, *r^2^*=0.20).


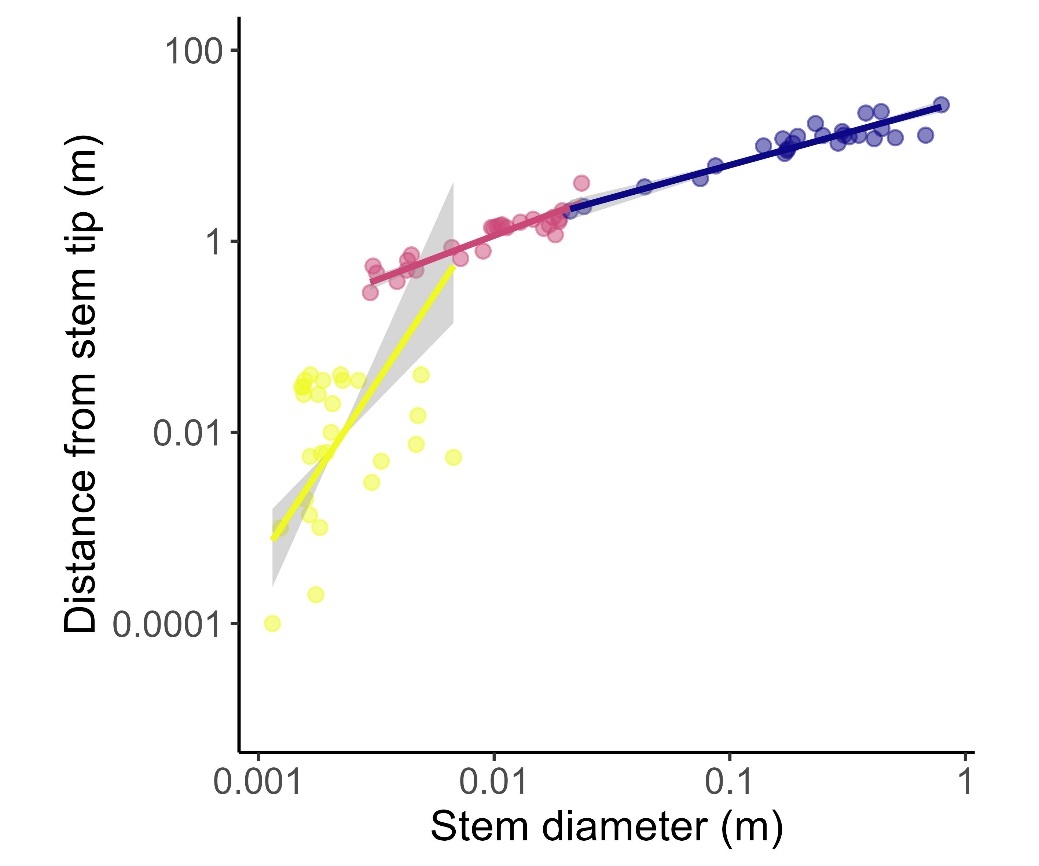


**Figure S15.** Relationship between external stem diameter *D* and distance from stem tip *L* fitted separately in twigs (slope = 3.74, 95% confidence interval (CI) = 2.53 – 5.52, *p* = 0.22, *r^2^* = 0.06; yellow points & line of best fit), branches (slope = 0.93, 95% CI = 0.80 – 1.09, *p* = 8.06 × 10^-12^, *r^2^*=0.85; dark pink & line of best fit), and main trunk (slope = 0.68, 95% CI = 0.58 – 0.79, *p* = 5.82 × 10^-12^, *r^2^*=0.87; dark violet points & line of best fit).


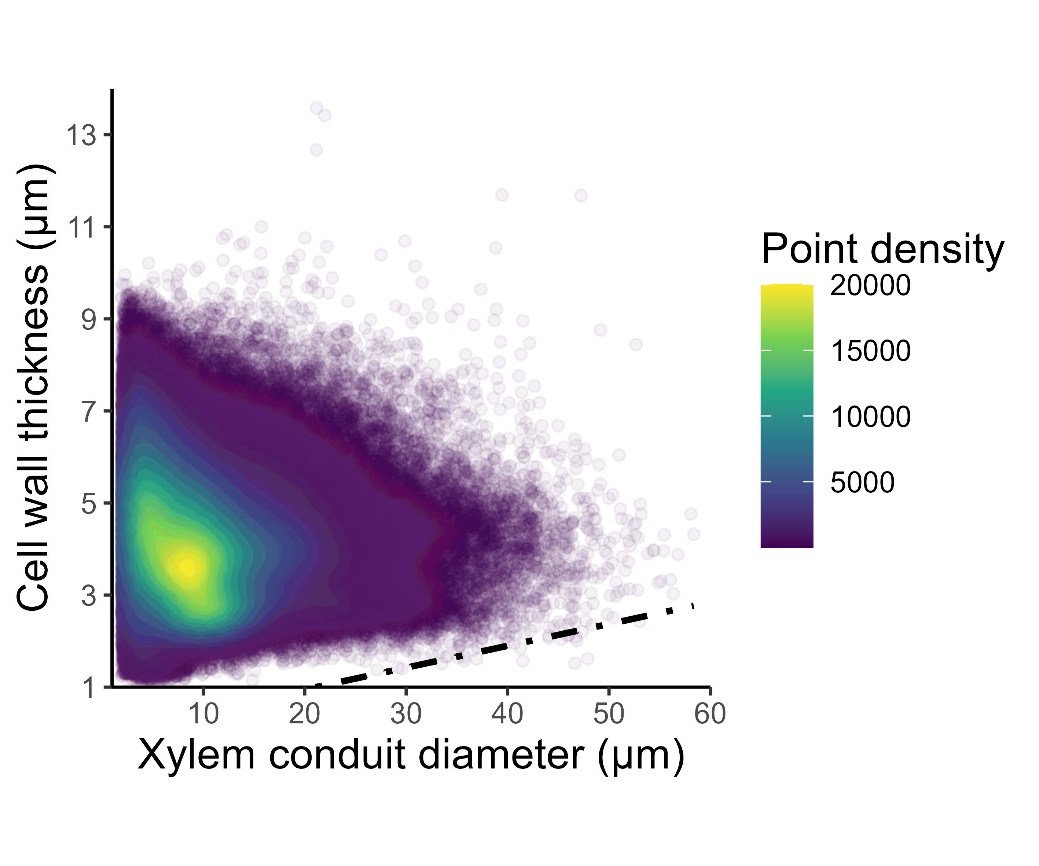


**Figure S16.** Relationship between mean single cell wall thickness (average of four single cell walls surrounding the conduit) and xylem conduit diameter in aboveground organs (i.e., leaves, twigs, branches, and trunks; *n* = 441,904). The dot-dashed line represents the lower threshold of the critical collapse limit (i.e., the critical collapse limit at the relative base of the hydraulic path, when *L* / *H* ≈ 100%). Many combinations of cell wall thickness and conduit diameter are observed except for wide conduits with thin cell walls (space represented by the bottom-right corner of plot, below the critical collapse limit). Wide conduits with very thick cell walls (upper right corner of the plot) are also relatively rare, possibly because the costs of constructing thick cell walls for a large conduit outweigh the benefits of being less vulnerable to collapse.
